# *Mori fructus* aqueous extracts attenuates liver injury by inhibiting ferroptosis via the Nrf2 pathway

**DOI:** 10.1186/s40104-023-00845-0

**Published:** 2023-04-10

**Authors:** Yuanyuan Wei, Chen Gao, Huiru Wang, Yannan Zhang, Jinhua Gu, Xiuying Zhang, Xuhao Gong, Zhihui Hao

**Affiliations:** 1 Innovation Centre of Chinese veterinary medicine, College of Veterinary Medicine, China Agricultural University, No. 2 Yuanmingyuan West Road, Beijing, 100193 China; 2 Key Biology Laboratory of Chinese Veterinary Medicine, Ministry of Agriculture and Rural Affairs, Beijing, 100193 P. R. China; 3 National Center of Technology Innovation for Medicinal function of Food, National Food and Strategic Reserves Administration, Beijing, China; 4 China Institute of Veterinary Drug Control, Beijing, 100081 China

**Keywords:** Acute and chronic liver injury, Ferroptosis, *Mori fructus* aqueous extracts, Nrf2, Oxidative stress

## Abstract

**Background:**

Liver fibrosis and hepatocellular carcinogenesis secondary to liver fibrosis are serious liver diseases with no effective treatments. *Mori fructus* aqueous extracts (MFAEs) have served as successful treatments for many types of liver injury including fibrosis although the molecular mechanisms are unknown at present.

**Purpose:**

To investigate the effect of MFAEs in alleviating acute and chronic liver injury and tried to decipher the underlying mechanism.

**Methods and results:**

Mice were divided into 5 groups (*n *= 8) for acute (groups: control, 0.3% CCl_4_, bifendate (BD), 100 and 200 mg/kg MFAEs, 7 d) and chronic (groups: control, 10% CCl_4_, BD, 100 and 200 mg/kg MFAEs, 4 weeks) liver injury study. Each mouse was injected intraperitoneally with 10 µL/g corn oil containing CCl_4_ expect the control group. HepG2 cells were used in vitro study. Eighteen communal components were identified by UPLC-LTQ-Orbitrap-MS. We utilized a mouse model for acute and chronic liver injury using CCl_4_ and MFAEs administration effectively blocked fibrosis and significantly inhibited inflammation in the liver. MFAEs activated the nuclear factor erythroid derived 2 like 2/heme oxygenase 1 (Nrf2/HO-1) pathway and promoted the synthesis of the antioxidants glutathione (GSH), superoxidedismutase (SOD) and glutathione peroxidase (GSH-Px) that resulted in reduced levels of CCl_4_-induced oxidative stress molecules including reactive oxygen species. These extracts administered to mice also inhibited ferroptosis in the liver by regulating the expression of Acyl-CoA synthetase long chain family member 4 (ACSL4), solute carrier family 7 member 11 (SLC7A11) and glutathione peroxidase 4 (GPX4), thus reducing the occurrence of liver fibrosis. Both in vivo and in vitro tests indicated that the mechanism of MFAEs protection against liver fibrosis was linked to activation of Nrf2 signaling. These effects were blocked in vitro by the addition of a specific Nrf2 inhibitor.

**Conclusion:**

MFAEs inhibited oxidative stress, ferroptosis and inflammation of the liver by activating Nrf2 signal pathway and provided a significant protective effect against CCl_4_-induced liver fibrosis.

**Graphical Abstract:**

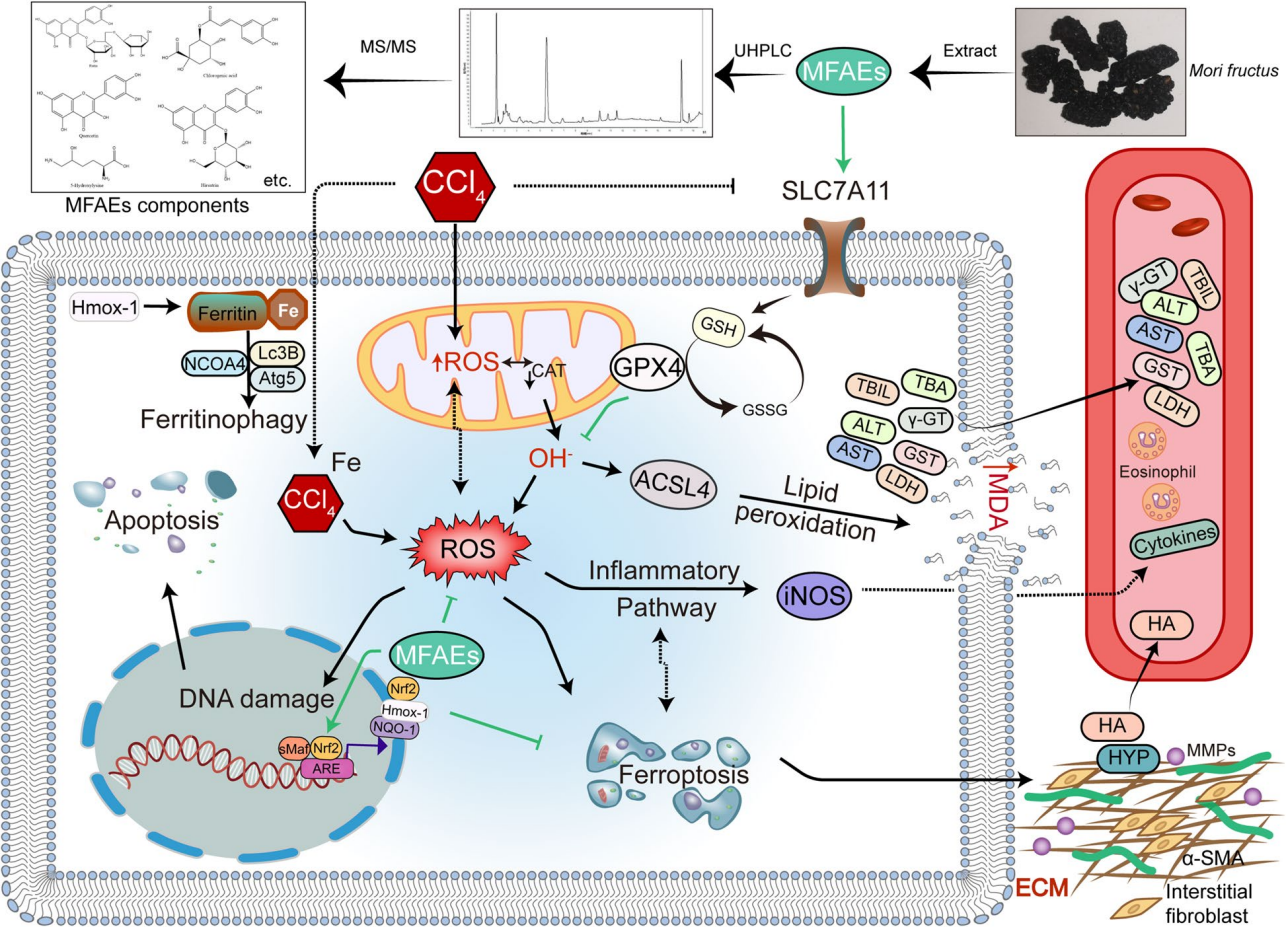

**Supplementary Information:**

The online version contains supplementary material available at 10.1186/s40104-023-00845-0.

## Introduction

Liver disease due to acute or chronic liver injury is a leading cause of human deaths worldwide [1, 2] even for children [3]. Hepatic fibrosis is a complication of liver injury and a common pathological result of long-term hepatic inflammation due to viral hepatitis, alcohol abuse, obesity-related nonalcoholic steatohepatitis (NASH) and autoimmune hepatitis lacking effective intervention. Untreated hepatic fibrosis will eventually progress into cirrhosis [4–6]. Importantly, the incidence of hepatocellular carcinoma (HCC) as a result of cirrhosis can reach levels as high as 90% and is a primary cause of death due to liver disease; the only effective treatment for fibrosis is liver transplantation [7, 8].

Treatments to prevent the progression of fibrosis to HCC have not been pharmacologically successful and the causes of fibrotic liver disease remain to be elucidated [9]. Ferroptosis has been linked to liver fibrosis and excess iron is a known risk factor and this has been directly demonstrated [10, 11]. The deposition of extracellular matrix materials by myofibroblasts derived from activated hematopoietic stem cells is the primary cause of hepatocirrhosis [12]. Additionally, alterations in the metabolism of stellate cell mitochondria can also lead to ferroptosis of these cells [13]. The links between ferroptosis and fibrosis are unclear at present although preventing ferroptosis is a viable clinical goal. Bifendate (or biphenyldicarboxylate, BD) is a synthetic intermediate of Schisandrin C. Bifendate is used clinically for the treatment of hepatitis with minimal observable side effects at the prescribed dose and is often used as a positive control to explore the effects of other hepatoprotective drugs [14]. It can reduce the CCl_4_-induced liver injury and transaminase levels [15]. Studies have demonstrated that *Schisandra chinensis* extracts including Schizandrin C exert multi-pharmaceutical bioactivities by activation of the Nrf2 mediated defense response [16].

Traditional Chinese medicines have been utilized to explore the therapeutics of liver fibrosis [17]. *Mori fructus* is the mature fruit of the mulberry tree that is globally distributed and is consumed as the fresh fruit, juice, jam and wine [18]. *M. fructus* has been used as a traditional Chinese drug to protect the liver and kidney and modern pharmacological studies have confirmed that this fruit possesses anti-oxidative properties and can reduce blood lipid levels [18–21]. The bioactive compounds in *M. fructus* aqueous extracts (MFAEs) have been shown to alleviate liver injury and preliminary results demonstrated a decrease in liver fibrosis although the physiological mechanisms for this response are not clear [22].

In the current study, we examined whether MFAEs can alter the course of hepatic fibrosis using in vitro cell cultures and an in vivo mouse model. Importantly, MFAEs were able to attenuate ferroptosis caused by experimental CCl_4_-induced fibrosis. These effects were linked to the increased expression of Nrf2 that is a central metabolic regulator especially for attenuating oxidative stress.

## Material and methods

### LC-MS conditions and chemical composition analysis

A Waters Aquity UPLC HSS T3 C18 (2.1 mm × 100 mm, 1.8 μm) chromatography column was utilized with mobile phases comprising: (A) 0.1% formic acid water and (B) acetonitrile. The gradient elution was as follows: 0–7 min (5%–9% B), 7–14 min (9%–15% B), 14–21 min (15%–20% B), 21–28 min (20%–60% B), 28–35 min (60%–95% B), and 35–42 min (95% B). The flow rate was 0.25 mL/min, the column oven temperature was 35 ℃, and the injection volume was 3 μL. HRMS spectral analysis was applied using an LTQ-Orbitrap Velos Pro mass spectrometer (Thermo Fisher Scientific, Waltham, MA, USA) equipped with an ESI source. The optimized operating parameters in the negative ion mode were: sheath gas flow rate of 30 arb, auxiliary gas flow rate of 10 arb, capillary voltage of −35 V, electrospray voltage of 3.0 kV, tube lens voltage of −110 V and capillary temperature of 300 °C. The constituents were determined using full-scan MS analysis from *m/z* 100 to 1500. Data-dependent ESI-MS analysis was triggered by the three most abundant ions.

An Xcalibur 2.2 workstation was employed for MS/MS data processing and analysis. Compound Discoverer 3.0 and Mzmine 2.53 software were used for background elimination, peak alignment, and common peak extraction of different quality spectrum data, and the common constituents of the three sources of MFAEs were tentatively determined. First, the data of known structural compounds were analyzed, and possible fracture mechanisms were predicted based on MS/MS fragments. The compounds were preliminarily identified by referring to the retention time and fragmentation pathway information provided by the PubChem, HMDB database, and references. The acceptable molecular mass error ≤ 5 ppm.

### Target prediction and pathway enrichment analysis

Active ingredients contained in MFAEs were selected according to the optimal toxicokinetic ADME (Absorption, Distribution, Metabolism, Excretion) rules for oral bioavailability, OB ≥ 30% and drug likeness, DL ≥ 0.18 obtained from the TCMSP (Traditional Chinese Medicine Systems Pharmacology) https://old.tcmsp-e.com/browse.php?qc=herbs (http://sm.nwsuaf.edu.cn/lsp/tcmsp.php). The targets of acute/chronic liver injury were obtained from the DisGeNET database (https://www.disgenet.org/) using the key words “hepatitis, chronic”, “acute hepatitis” and “fibrosis, liver” and scores were filtered as gda ≥ 0.3. Venn maps were constructed using Jven (http://jvenn.toulouse.inra.fr/app/example.html) using the overlapped targets MFAEs and acute/chronic liver injury [23]. The Reactome Pathway online database (https://reactome.org/PathwayBrowser/) was used for pathway enrichment analysis and the top 20 items were illustrated using the R platform (4.2.1) [24] and Cytoscape 3.9.1 [25].

### Cell culture

HepG2 cells were purchased from the National Cell Resource Sharing Platform (Beijing, China) and incubated in MEM containing 10 % fetal bovine serum and 1% penicillin/streptomycin (Gibco Laboratories, Shanghai, China) in a 5% CO_2_ humidified atmosphere at 37 ℃. The cells were passaged using 0.05% trypsin/EDTA in 6- and 24-well plates and cultured for 24 h. MFAEs were added at 20 µg/mL for 12 h and the cells were then stimulated with 20 mmol/L CCl_4_ or 10 µmol/L Nrf2 inhibitor ML385 (MedChemExpress, Grangetown, Cardiff, Monmouth, UK; Cat. No. HY-100523, CAS: 846557-71-9) for 6 h before harvest.

### Reactive oxygen species (ROS) analysis

Intracellular ROS production in culture cells was measured using a commercial kit; Reactive Oxygen Species Assay Kit (Solarbio, Beijing, China; Cat. No. CA1410) according to the manufacturer’s instructions. In brief, HepG2 cells were seeded in 24-well plates at 10^4^ cells/mL and incubated with 10 µmol/L DCFH-DA for 15 min at 37 ℃ and conversion to DCFH was tracked using an inverted fluorescence microscope (Olympus, Tokyo, Japan, CKX53).

### FITC annexin V apoptosis detection

HepG2 cells were seeded in 6-well plates and incubated with FITC and PI for 15 min, respectively, followed by PBS washing. Apoptotic cells were detected using a CytoFlex flow cytometer (Beckman Coulter, Brea, CA, USA), which pre-treated with a commercial FITC Annexin V Apoptosis Detection Kit (BD Biosciences, Franklin Lakes, NJ, USA; Cat. No. 556547).

### Fe^2+^ staining assays

HepG2 cells were seeded at 5 × 10^4^ into 24-well plates and pre-incubated for 12 h with 20 μg/mL MFAEs or vehicle in the absence and presence of 20 mmol/L CCl_4_ or 10 µmol/L ML385 for 6 h. The cells were stained using 1 μmol/L Ferro orange (Dojingo, Rockville, MD, USA; Cat. No. F374) and 10 μg/mL DAPI in HBSS for exactly 30 min at 37 °C and 5% CO_2_. The cells were then immediately imaged. Treatments were staggered to ensure precise staining durations and images were acquired using an SP8 laser scanning confocal microscope (Leica, Wetzlar, Germany) using a 63×/1.4 DIC Plan-Apochromat oil immersion objective. Images were obtained using the Cy3 filter with excitation at 514 and emission at 525–596 nm as previously described [26].

### Lactoperoxidase (LPO) observation

HepG2 cells were seeded at 3 × 10^4^ in 6-well plates and incubated overnight at 37 ℃ in a 5% CO_2_ incubator. Cells were incubated with 1 µmol/L Liperfluo (Dojingo, Rockville, MD, USA; Cat. No. L248) for 30 min as per above after washing with MEM medium. The solution was discarded and the cells were washed with PBS and 200 µL 500 µmol/L *tert*-butyl hydroperoxide was then added and the cells were incubated for 60 min and observed under a Leica SP8 confocal microscope and images were analyzed and merged using the LAS AF Lite software supplied with the instrument.

### Animals

Eight-week-old male ICR mice (18 ± 2 g) were purchased from Speyford Experimental Animal Science and Technology (Beijing, China). All animals were maintained in a barrier environment under a 12 h light/dark cycle, at a temperature of 23 ± 1 ℃, and 50%–60% humidity with free access to pelletized chow and water throughout the entire experiment.

### Experimental design

**Fig. 1:**
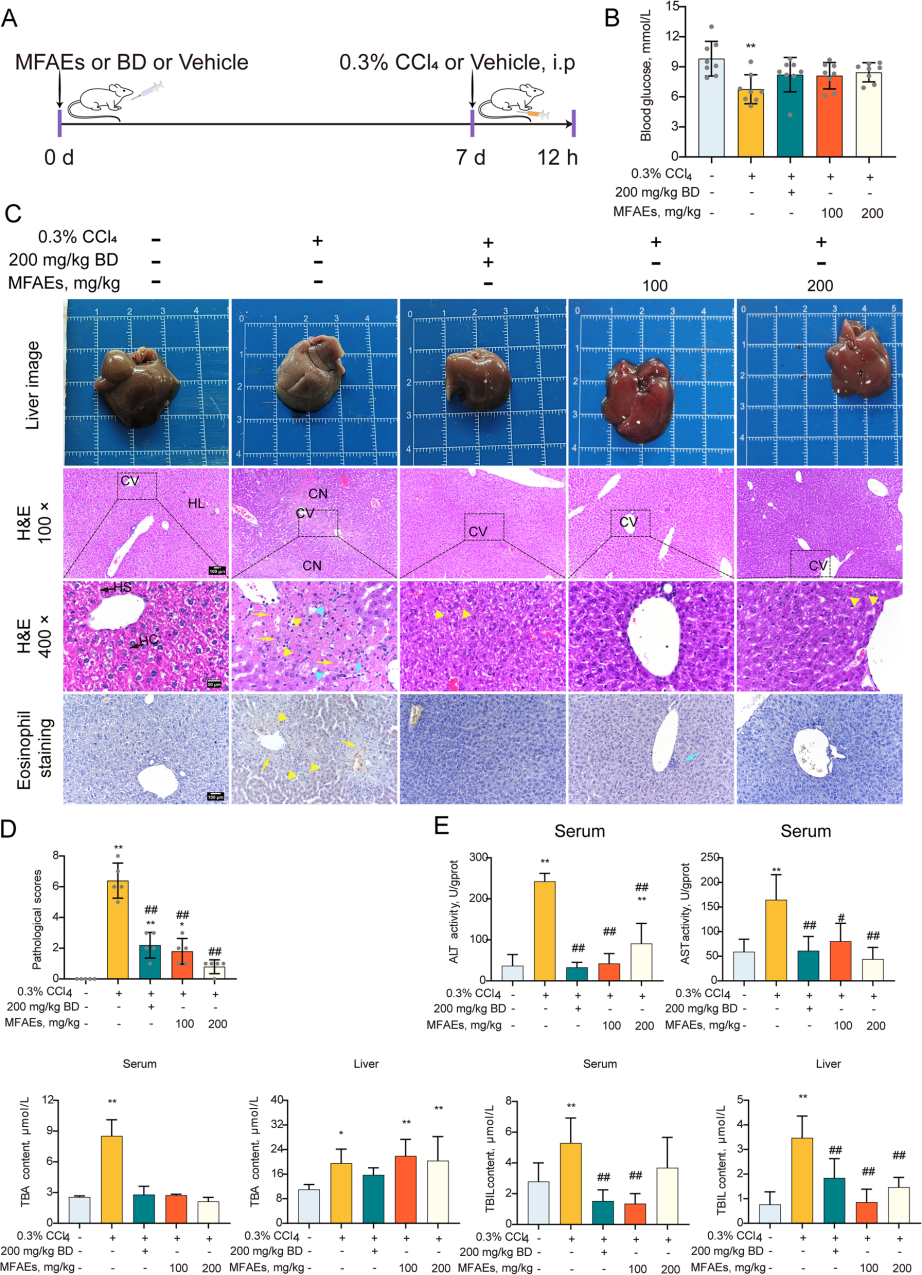
MFAEs restored hepatic function and structure in CCl_4_-induced acute liver injury mice. **A** Outline of experimental procedures for CCl_4_-induced acute liver mouse model. **B** Blood glucose. *n* = 8 samples per group. **C** Representative liver image, H&E and eosinophil staining images of liver sections as indicated. Bar, 100 μm (100× and 200×), 50 μm (400×). *n *= 5 per group. CN: Coagulative necrosis, HL: Hepatic lobule, HS: Hepatic sinusoid, HC: Hepatic cord. Yellow arrow: hepatocyte edema, Blue arrow: inflammatory cell infiltration, yellow triangle: eosinophilic corpuscle. Blue triangle: lipid degeneration. **D** Pathological scores. **E** Serum ALT and AST activity, serum and liver tissue TBIL and TBA contents in the indicated mice. Values are expressed as mean ± SD. One-way ANOVA with Tukey’s analysis was used. *Indicates a significant difference compared with the control group (*P < 0.05, **P < 0.01). ^#^Indicates a significant difference compared with the 0.3% CCl_4_ group (^#^*P* < 0.05, ^##^*P* < 0.01)

Mice were divided into 5 groups (*n *= 8) as follow: control group, model group, positive drug group, low-dose MFAEs group, and high-dose MFAEs group. For acute liver injury model, control and model mice were perfused with 0.2 mL distilled water for 7 d. Mice in groups positive drug, low-dose MFAEs and high-dose MFAEs were administered 200 mg/g BD, 100 and 200 mg/kg MFAEs orally once a day through the 7 d. On d 7, mice in control group were injected intraperitoneally (i.p) with corn oil (10 µL/g) , mice in other four groups were injected with corn oil (10 µL/g) containing 0.3% CCl_4_. All mice were sacrificed 12 h later using sodium pentobarbital (Fig. 1A).

For chronic liver injury, controls were perfused with 0.2 mL distilled water for 4 weeks and treated with corn oil (with or without 10% CCl_4_, see above) at d 1, 3 and 5 of the week. The mice were given 100 and 200 mg/kg MFAEs orally for 4 weeks followed with 10% CCl_4_ in low-dose and high-dose MFAEs groups [27]. Positive drug group were given 200 mg/g BD at the same days (Fig. 4A).

### Analysis of blood glucose and liver index

Body weights was monitored and livers of the mice were dissected, weighed and directly placed at −80 ℃. Liver indices were measured as liver weight (g)/body weight (g) × 100%. Blood for glucose measurements were taken by tail vein sampling. The serum was obtained from the supernatant after centrifugation at 1500 × *g* for 15 min.

### Histological analysis

Liver tissues were separated from each sample and fixed in 4% formaldehyde for 1 week and then embedded in paraffin, followed by slicing into 4 μm sections and staining with hematoxylin and eosin (H&E) and a commercial eosinophil Sirius Red staining kit (Solarbio, Beijing, China, Cat. No. G3632) and Masson trichrome fast green stain kit (Solarbio, Beijing, China, Cat. No. G1343) according to manufacturer’s procedures, respectively. Images were obtained by light microscopy (DS-Ri2, Nikon, Tokyo, Japan). Five different visual fields were observed for each section and graded with reference to the Ishak scoring rules (Tables S1 and S2).

### Cytokine measurement

Liver homogenates were prepared and centrifuged as previously described [28, 29]. The liver homogenate supernatants and serum samples were used for cytokine measurements using commercial ELISA kits (Elabscience, Houston, TX, USA) as follows: Interleukin 1 beta (IL-1β; Cat. No. E-EL-M0037c), Interleukin 6 (IL-6; Cat. No. E-EL-M0044c) and TNF-α (Cat. No. E-EL-M3063).

### Serum assay and hepatic function assay

Serum samples were also used to measure alanine aminotransferase (GPT/ALT; Cat. No. C009-2-1), aspartate aminotransferase (GOT/AST; Cat. No. C010-2-1), and gamma-glutamyltransferase (γ-GT; Cat. No. C017-2-1). Lactate dehydrogenase (LDH; Cat. No. A020-2-2), total bile acid (TBA; Cat. No. E003-2-1) and total bilirubin (TBIL; Cat. No. C019-1-1) levels in serum and liver homogenates were detected using commercial kits (Nanjing Jiancheng Bioengineering, Nanjing, China).

### Hepatic fibrosis and oxidation parameters detection

The levels of hydroxyproline (HYP; Cat. No. A030-2-1) and hyaluronic acid (HA; Cat. No. H141-1-2) in liver homogenate were determined using commercial kits (see below) using an Infinite M200Pro Nanoquant Instrument (Tecan, Mannedorf, Switzerland). Enzyme activity levels of total superoxide dismutase (T-SOD; Cat. No. A001-1), catalase (CAT; Cat. No. A007-1-1), total antioxidant capacity (T-AOC; Cat. No. A015-2-1), malondialdehyde (MDA; Cat. No. A003-1-2), peroxidase (POD; Cat. No. A084-1-1), glutathione S-transferase (GST; Cat. No. A004-1-1), glutathione (GSH; Cat. No. A006-2-1) and glutathione peroxidase (GSH-px; Cat. No. A005-1-1) in liver and serum were determined using commercial kits (Nanjing Jiancheng Bioengineering, Nanjing, China).

### Western blot assay

Total proteins in liver tissues or HepG2 cells were obtained using total protein extraction (Solarbio, Beijing, China; Cat. No. BC3710) and nuclear protein extraction (Solarbio, Beijing, China; Cat. No. R0050) kits and quantified using a BCA Protein Assay Kit (Thermo Fisher Scientific, Waltham, MA, USA; Cat. No. A53225). Equal amounts of protein were separated using 12% SDS–PAGE and electrotransferred to PVDF membranes (240 mA, 90 min) and probed with primary antibodies for 8 h at 4 ℃ (Table S3). HRP-conjugated Affinipure Goat Anti-Rabbit IgG (H+L) (Proteintech, Wuhan, China; Cat. No. SA00001-2, 1:5000) was used as the secondary antibody. The membranes were washed with TBST for 7 min × 5 times followed by detection using the ECL Prime Western Blotting Detection Reagent (Cytiva, Marlborough, MA, USA; Cat. No. RPN2232). Band intensities were analyzed as previously described [30].

### Immunohistochemical staining

Paraffin sections were blocked with PBS containing 5% BSA for 2 h at 37 ℃ and incubated with primary antibodies (Table S4) at 37 ℃ for 2 h and then washed and incubated with a matching secondary antibody for 2 h, at 37 ℃. Results were visualized using DAB staining (Solarbio, Beijing, China; Cat. No. DA1010) and examined by light microscopy (DS-Ri2).

### Transferase dUTP nick end labeling (TUNEL) assay

Liver sections were prepared and dewaxed according to the manufacturers protocol (Boster, Pleasanton, CA, USA; Cat. No. MK1025). In brief, 3% H_2_O_2_ was used to eliminate endogenous peroxidase and proteinase K was used to permeate the cells at 37 ℃ for 15 min following by TBS washing for 2 min × 3 times. TDT and DIG-d-UTP (1:100) were added and incubated at 37 ℃ for 2 h and was with TBS 2 min × 3 times. The sections were blocked for 30 min at 37 ℃ and then were incubated with biotinylated anti-digoxin antibody at 37 ℃ for 2 h following by SABC incubation. The results were visualized using DAB (Solarbio, Beijing, China; Cat. No. DA1010).

### RNA extraction, reverse transcription and q-PCR

Total RNA was extracted from the liver tissue using a total RNA extraction kit (Promega, Madison, WI, USA; Cat. No. LS1040). Single-strand cDNA was reverse-transcribed from the extracted RNA using the GoScript Reverse Transcription System (Thermo Fisher Scientific, Waltham, MA, USA; Cat. No. K1622) following the manufacturer’s instructions and qPCR was conducted on a CFX96 Touch Real-Time PCR Detection System (Bio-Rad, Hercules, CA, USA). Amplification was performed for 45 cycles consisting of 5 s at 95 ℃, and 30 s at 64 ℃, then 30 s at 72 ℃. Gene expression was quantified relative to the internal control gene *Gapdh* using the comparative Ct method and expressed as relative mRNA level (Table S5).

### Statistical analysis

Statistical analyses were performed using IBM SPSS Statistics 23.0. All results are expressed as the mean ± SD and statistical significance was tested by one-way ANOVA and followed with Tukey’s multiple comparison procedure. For two groups, statistical significance was determined by independent sample *t*-test. *P* values are specified in the figure legends.

## Results

### Chemical composition identification in MFAEs and potential mechanism predication of alleviating liver injury

To determine chemical ingredients in MFAEs, sample analysis was performed in the negative ion mode. After subtracting blank and removing peak adducts that were generated in source, the compounds were tentatively identified from their exact mass, isotope patterns, and mass fragmentation patterns. A total of 18 communal compounds were preliminarily identified by LC-Orbitrap-ESI-MS (Table S6, Fig. S1). We further collected potential targets for MFAEs effects on acute/chronic liver injury in TCMSP, DisGent and OMIM databases and gained 13 overlapped targets using the Venn tools (Fig. S2A). The 13 overlapped targets [NFE2L2, HO-1, Vascular endothelial growth factor A (VEGFA), Interleukin 6 (IL-6), SOD1, Matrix metallopeptidase 2 (MMP2), Chemokine (C-C motif) ligand 2 (CCL2), Coagulation factor III (F3), Cytochrome P450, family 1, subfamily a, polypeptide 2 (CYP1A2), Collagen, type I, alpha 1 (COL1A1), Aryl-hydrocarbon receptor, Coagulation factor II (F2) and urokinase-type plasminogen activator (PLAU)] were linked to GO: 0034440: lipid oxidation, GO: 0048145: regulation of fibroblast proliferation, GO: 0038061: NIK/NF-κB signaling, HSA-449147: Signaling by Interleukins 6, WP3888: VEGFA-VEGFR2 signaling pathway and WP408: Oxidative stress response (Fig. S2B and C).

### MFAEs restored hepatic function and structure in CCl_4_-induced acute liver injury mice

We further measured hepatic function in our acute liver injury model. Blood glucose levels in the 0.3% CCl_4_ group were markedly reduced compared with controls (*P* < 0.01) (Fig. 1B). Histology and eosinophil staining indicated necrosis and inflammatory cell infiltration in hepatocytes treated with CCl_4,_ especially eosinophils, while the number of necrotic and infiltrating inflammatory cells in the livers of MFAEs-treated mice decreased significantly (Fig. 1C and D). MFAEs were also able to inhibit the increase of the biomarkers of CCl_4_-induced liver injury reflected in decreases of ALT and AST in serum and TBA and TBIL in serum and liver tissues (*P* < 0.01) (Fig. 1E).

### MFAEs attenuated fibrosis and inflammation in CCl_4_-stimuated acute liver injury mice

**Fig. 2:**
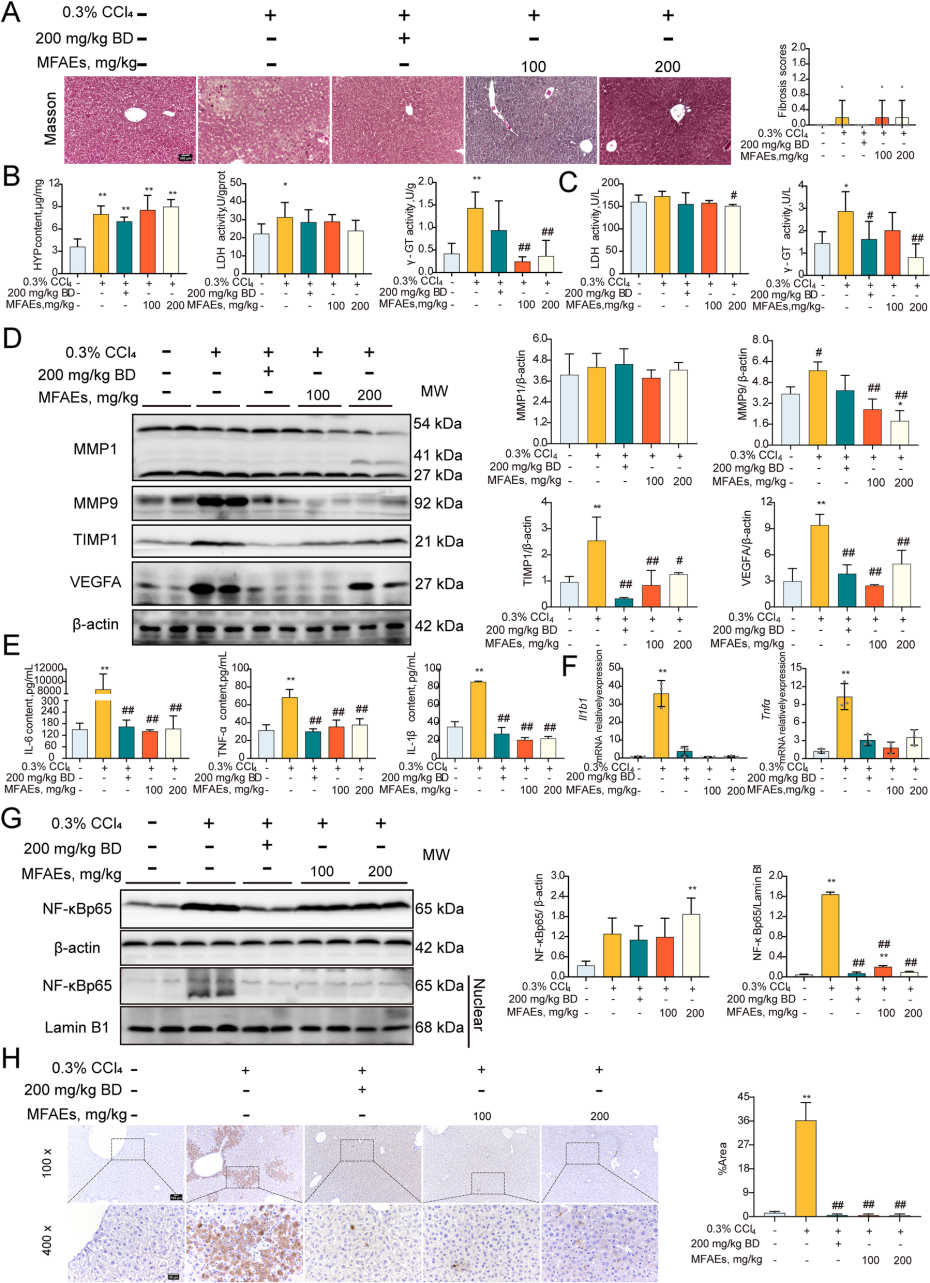
MFAEs attenuated fibrosis and inflammation in CCl_4_-induced acute liver injury mice. **A** Masson staining images of liver sections and fibrosis scores in indicated mice. Bar, 100 μm. Black arrow: congestion, yellow arrow: hepatocyte edema, red triangle: positive area. **B** Liver HYP, LDH and γ-GT levels in indicated mice. **C** Serum LDH and γ-GT levels in indicated mice. **D** Western blot analysis of MMP1, MMP9, TIMP1 and VEGFA in the livers of indicated mice. β-actin served as loading control. *n *= 3 per group. **E** Serum IL-6, TNF-α and IL-1β content in indicated mice. *n *= 6 per group. **F** Relative mRNA levels of *Tnf* and *Il1b*. **G** Western blot analysis of total and nuclear NF-κBp65 in the livers of indicated mice. Lamin B1 and β-actin served as loading controls. *n *= 3 per group. **H** TUNEL analysis in the livers of indicated mice. Bar, 100 μm (100×), 50 μm (400×). *Indicates a significant difference compared with the control group (*P < 0.05, **P < 0.01). ^#^Indicates a significant difference compared with the 0.3% CCl_4_ group (^#^*P* < 0.05, ^##^*P* < 0.01)

Liver fibrosis is the primary manifestation of liver injury. In our acute mouse model, Masson staining indicated no significant differences in the degree of liver fibrosis among our 2 experimental and one control group (*P* > 0.05) (Fig. 2A). MFAEs significantly inhibited HYP appearance in serum and increased LDH and γ-GT in liver and serum vs. controls (*P* < 0.01) (Fig. 2B and C). Immunoblotting results revealed that the expression of fibrosis-associated factors could be significantly decreased by MFAEs. These included MMP1, MMP9, Metallopeptidase inhibitor 1 (TIMP1) and VEGFA and these levels were consistent with the immunohistochemistry results (*P* < 0.01) (Fig. 2D, S2A).

Inflammation plays an important role in promoting liver fibrosis so we measured proinflammatory cytokine expression. Interleukin 1 beta (IL-1β), IL-6 and tumor necrosis factor alpha (TNF-α) in serum and the mRNA levels of *Il1b1* and *Tnf-α* were strikingly reduced following 100 or 200 mg/kg MFAEs administration (*P* < 0.01) (Fig. 2E). The cytokine content for these in liver were not significantly different among the 3 groups (*P* > 0.05) (Fig. S3). MFAEs also significantly inhibited the expression of the p65 protein of the NF-κB pathway in the nucleus indicating that MFAEs produced an anti-inflammatory effect (*P* < 0.01) (Fig. 2G). In addition, MFAEs effectively prevented excessive apoptosis of liver cells (*P* < 0.01) (Fig. 2H).

### MFAEs boosted antioxidant capacity in CCl_4_-induced acute liver injury mice

**Fig. 3:**
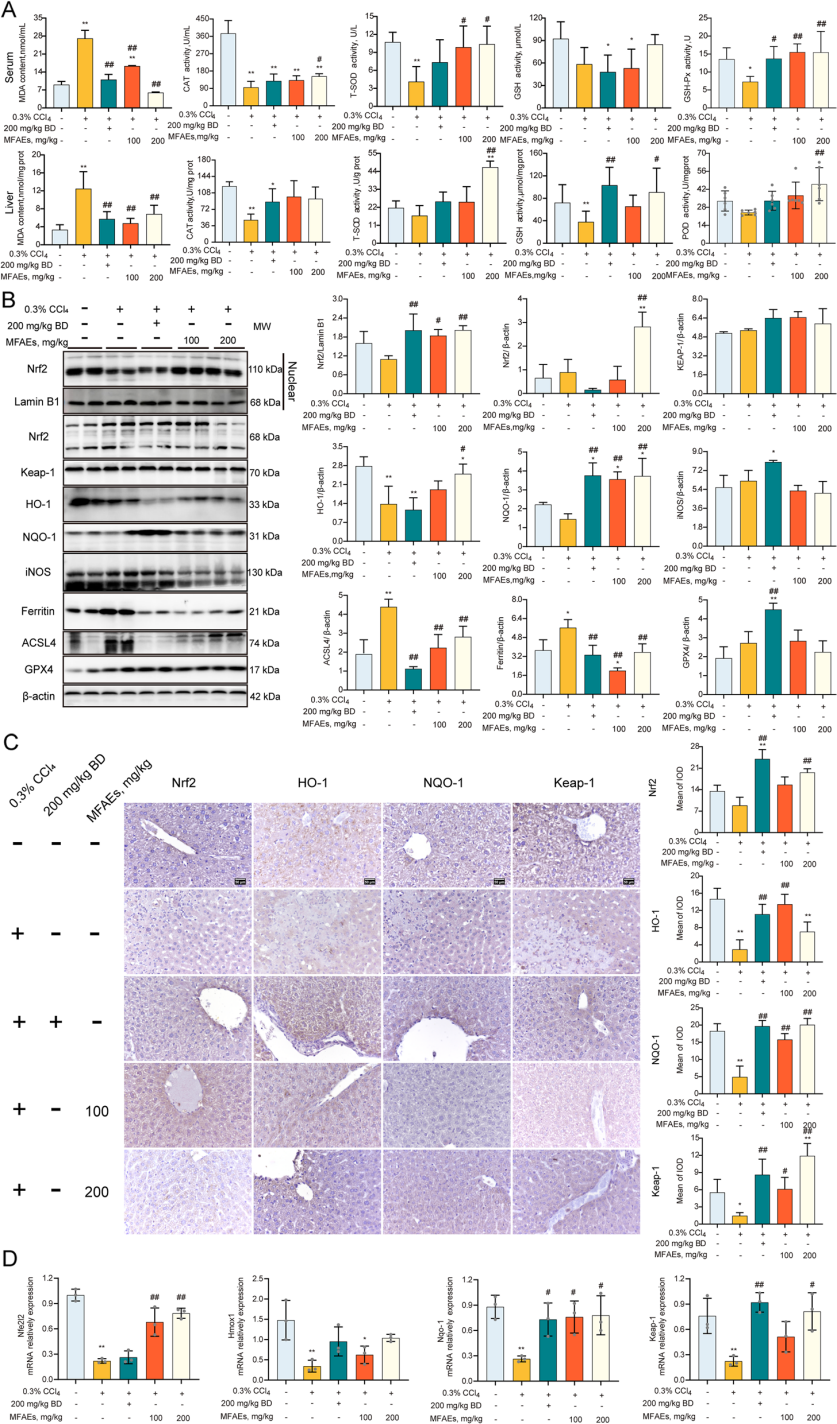
MFAEs boost antioxidant capacity in CCl_4_-induced acute liver injury mice. **A** Serum and liver antioxidants levels of indicated mice. **B** Western blot analysis of total and nuclear Nrf2, Keap-1, HO-1, NQO-1, iNOS, Ferritin, ACSL4 and GPX4 in the livers of indicated mice. Lamin B1 and β-actin served as loading control. **C** Representative image of immunohistochemical staining for Nrf2, HO-1, NQO-1 and Keap-1 in the livers of indicated mice. Bar, 50 μm. Black arrow: congestion, yellow arrow: hepatocyte edema, red triangle: positive area. **D** Relative mRNA levels of *Nfe2l2*, *Hmox-1*, *Nqo-1* and Keap-1. *Indicates a significant difference compared with the control group (*P < 0.05, **P < 0.01). ^#^Indicates a significant difference compared with the 0.3% CCl_4_ group (^#^*P* < 0.05, ^##^*P* < 0.01)

To gain insights into the mechanism of MFAEs regulation in CCl_4_-stimulated acute liver injury mice, we assayed oxidative stress and ferroptosis pathways. MFAEs administration to mice significantly inhibited MDA production in liver and serum of mice and significantly increased the activity of the antioxidant factors CAT, T-SOD, GSH-px, GSH and POD (*P* < 0.01) (Fig. 3A). MFAEs also significantly increased Nrf2, HO-1 and NQO-1 protein levels and mRNA accumulation for the Keap1-Nrf2 pathway members (*P* < 0.01). Immunohistochemical staining indicated expression of Nrf2, HO-1 and NQO-1 in the liver were significantly increased after MFAEs treatment (*P* < 0.01) (Fig. 3B and C). In addition, MFAEs also significantly inhibited iNOS expression in the liver (*P* < 0.01). Expression of ferritin and ACSL4 levels were significantly lower in mice administered MFAEs while GPX4 protein levels were stabilized (*P* < 0.01) (Fig. 3B–D). These results indicated that MFAEs can significantly alleviate oxidative stress and ferroptosis in CCl_4_-induced acute liver injury.

### MFAEs improved hepatic function and structure in CCl_4_-induced chronic liver injury mice

**Fig. 4:**
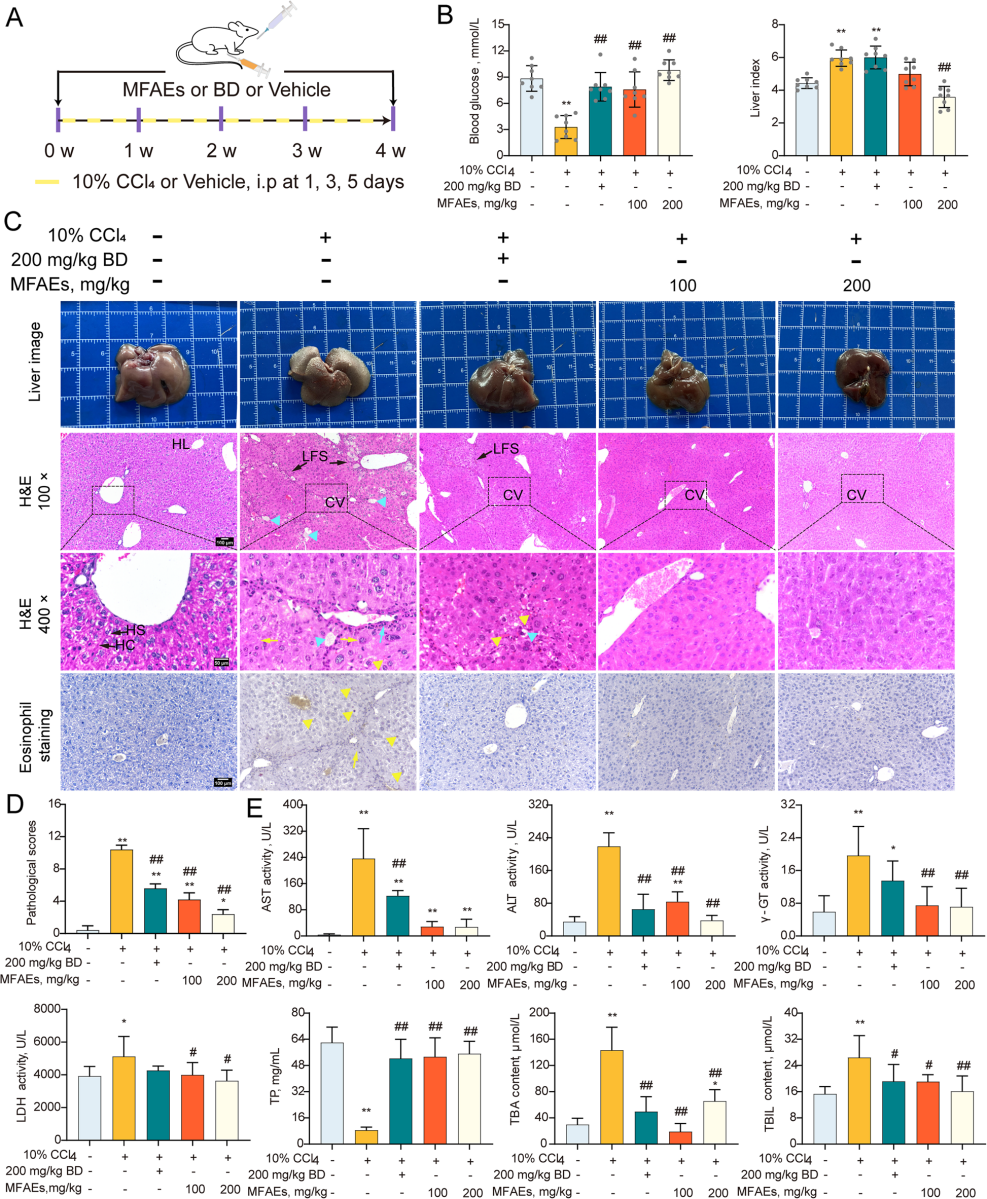
MFAEs improve hepatic function and structure in CCl_4_-induced chronic liver injury mice. **A** Outline of experimental procedures to examine the role of MFAEs in the mouse 10% CCl_4_-induced chronic liver model. **B** Blood glucose and liver indices. **C** Representative liver image, H&E and eosinophil staining images of liver sections in indicated mice. Bar, 100 μm (100× and 200×), 50 μm (400×). *n *= 5 per group. LFS: Long fiber spacing, HL: Hepatic lobule, HS: Hepatic sinusoid, HC: Hepatic cord. Yellow arrow: hepatocyte edema, blue arrow: inflammatory cell infiltration, yellow triangle: eosinophilic corpuscle. **D** Pathological scores. **E** Serum ALT, AST, γ-GT and LDH activity, serum TP, TBIL and TBA contents in the indicated mice. *Indicates a significant difference compared with the control group (*P < 0.05, **P < 0.01). ^#^Indicates a significant difference compared with the 10% CCl_4_ group (^#^*P* < 0.05, ^##^*P* < 0.01)

We further explored the impact of MFAEs on hepatic functions and structure in chronic liver injury caused by CCl_4_. MFAEs blocked the decrease of blood glucose and the increase of liver coefficient induced by 10% CCl_4_ in mice (*P* < 0.01) (Fig. 4B). The overall appearance of the livers in MFAEs-treated mice were also improved. Furthermore, MFAEs effectively decreased LFS broadening, pseudolobule formation, cellular lipid degeneration and eosinophil infiltration that are indicative of CCl_4_ injury (Fig. 4C and D). The mice given MFAEs also displayed lower levels of serum AST, ALT, TBA, TBIL, γ-GT and LDH and TP induced by CCl_4_ in mice (*P* < 0.01 or *P* < 0.05) (Fig. 4E).

### MFAEs suppressed fibrosis and inflammation in CCl_4_-induced chronic liver injury mice

**Fig. 5:**
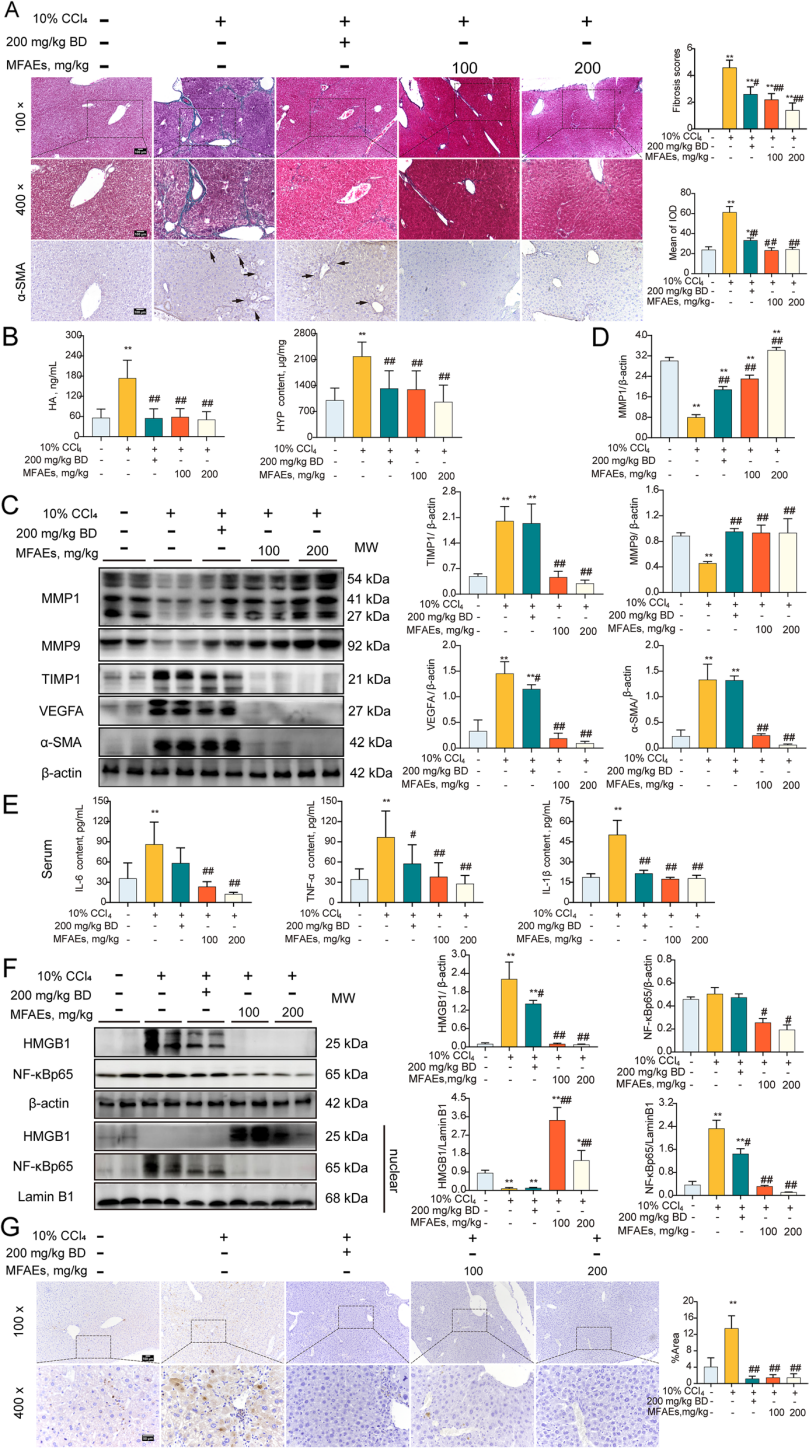
MFAEs suppresses fibrosis and inflammation in CCl_4_-induced chronic liver injury mice. **A** Masson staining and immunohistochemistry staining for α-SMA images of liver sections and fibrosis scores in the indicated mice. Bar, 100 μm. Black arrow: pseudolobule, yellow arrow: fibrosis, red triangle: positive area. **B** Serum HA and liver HYP contents in indicated mice. **C** Western blot analysis of MMP1, MMP9, TIMP1, VEGFA and α-SMA in the livers of indicated mice. β-actin served as loading control. **D** Semiquantitative analysis. **E** Serum IL-6, TNF-α and IL-1β in indicated mice. **F** Western blot analysis of total and nuclear HMGB1 and NF-κBp65 in the livers of indicated mice. Lamin B1 and β-actin served as loading controls. **G** TUNEL analysis in the livers of indicated mice. Bar, 100 μm (100×), 50 μm (400×). *Indicates a significant difference compared with the control group (*P < 0.05, **P < 0.01). ^#^Indicates a significant difference compared with the 10% CCl_4_ group (^#^*P* < 0.05, ^##^*P* < 0.01)

Given the benefit of MFAEs in maintaining liver function and structure in acute liver model, we analyzed fibrosis and inflammation in a chronic liver model. MFAEs could significantly alleviate liver fibrosis and significantly reduced the distribution of alpha-smooth muscle actin (α-SMA) protein in liver tissue and HA and HYP level in serum (*P* < 0.01) (Fig. 5A and B). Similarly, all dosages of MFAEs significantly increased the expression of MMP1 and MMP9 in the liver induced by CCl_4_ and inhibited the overexpression of TIMP1, VEGFA and α-SMA in the liver (*P* < 0.01) (Fig. 5C, D and S2C). In addition, inflammatory cytokines IL-1β, IL-6 and TNF-α levels in serum were repressed by MFAEs treatment in a dose-dependent manner (*P* < 0.01) (Fig. 5E). Protein levels of high mobility group box 1 (HMGB1) and p65 in the nucleus were also significantly reduced by MFAEs administration (*P* < 0.01) (Fig. 5F). Hepatocyte apoptosis was also significantly inhibited by MFAEs suggesting that these compounds inhibit inflammation to reduce fibrosis in the livers of mice treated with CCl_4_ (*P* < 0.01) (Fig. 5G).

### MFAEs weakened ferroptosis through antioxidant system in CCl_4_-induced chronic liver injury mice

**Fig. 6:**
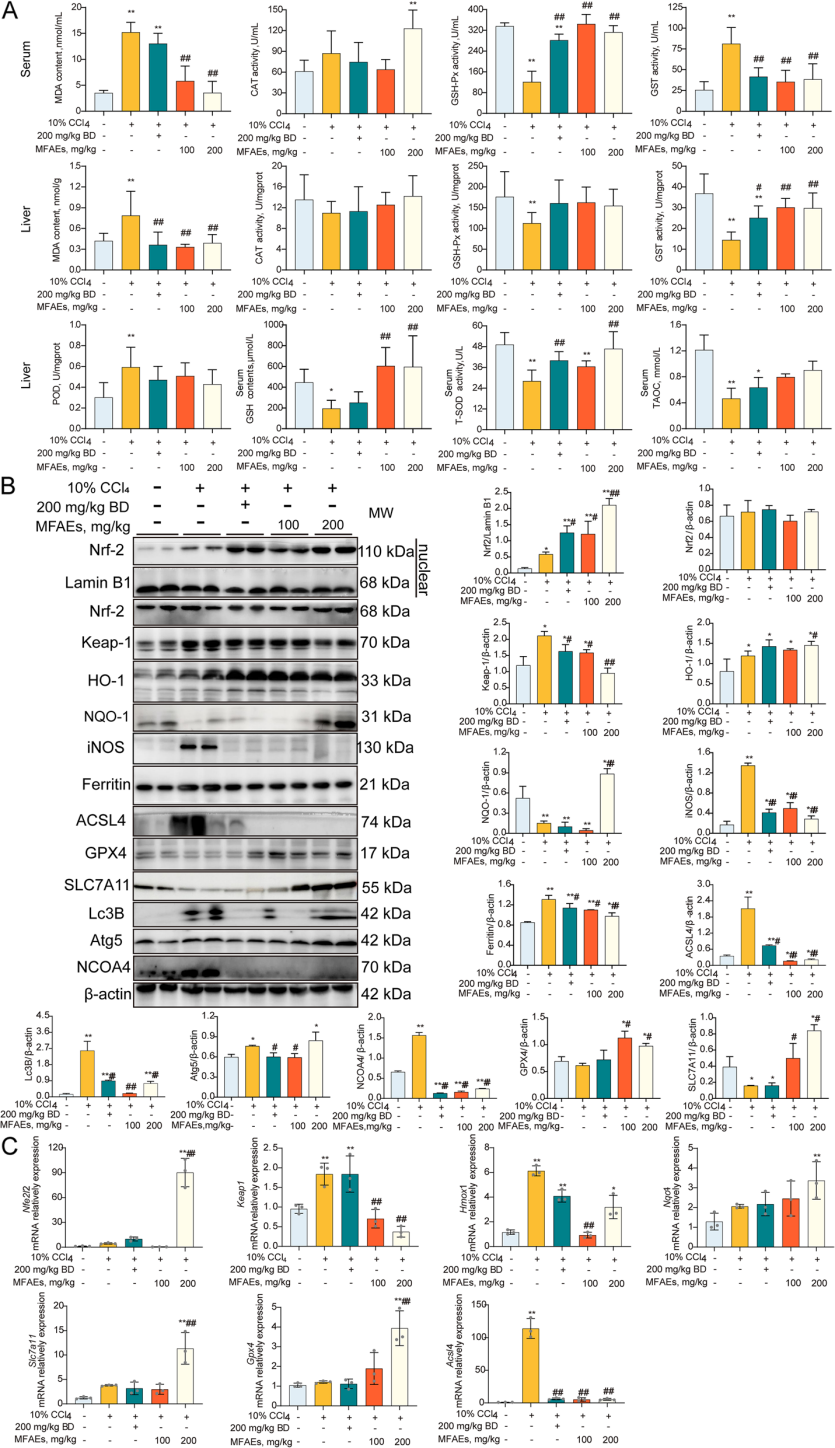
MFAEs weakened ferroptosis through antioxidant system in CCl_4_-induced chronic liver injury mice. **A** Serum and liver antioxidant levels of indicated mice. **B** Western blot analysis of total and nuclear Nrf2, Keap-1, HO-1, NQO-1, iNOS, Ferritin, ACSL4, GPX4, SLC7A11, Lc3B, Atg5 (autophagy related 5) and NCOA4 in the livers of indicated mice. Lamin B1 and β-actin served as loading control. **C** Relative mRNA levels of *Nfe2l2*, *Hmox-1*, *Nqo-1*, *Keap-1*, Slc7a11, *Gpx4* and *Acsl4.* Results are expressed as mean ± SD. *Indicates a significant difference compared with the control group (*P < 0.05, **P < 0.01). ^#^Indicates a significant difference compared with the 10% CCl_4_ group (^#^*P* < 0.05, ^##^*P* < 0.01)

To test the hypothesis whether MFAEs reverse chronic liver injury via the Nrf2 pathway, antioxidant enzymes and protein expression in the liver were measured. MFAEs significantly reduced MDA and increased CAT (catalase) and GSH-px levels in serum (*P* < 0.01). Similarly, MDA levels in liver tissue were decreased while GST, GSH and T-SOD were increased with MFAEs administration (*P* < 0.01) (Fig. 6A).

The Nrf2 pathway is an important oxidative stress inhibitory pathway in vivo. We found that both protein and mRNA expression levels of key factors (Nrf2, HO-1 and NQO-1) in Nrf2 pathway and related ferroptosis inhibitory factors (SLC7A11 and GPX4) were significantly increased by MFAEs administration (*P* < 0.01) (Fig. 6B, C and S2D). Expression levels of Lc3B, NCOA4 and iNOS were also significantly diminished with MFAEs administration (*P* < 0.01) (Fig. 6B).

### MFAEs attenuated Fe^2+^ accumulation and inflammation in vitro

**Fig. 7:**
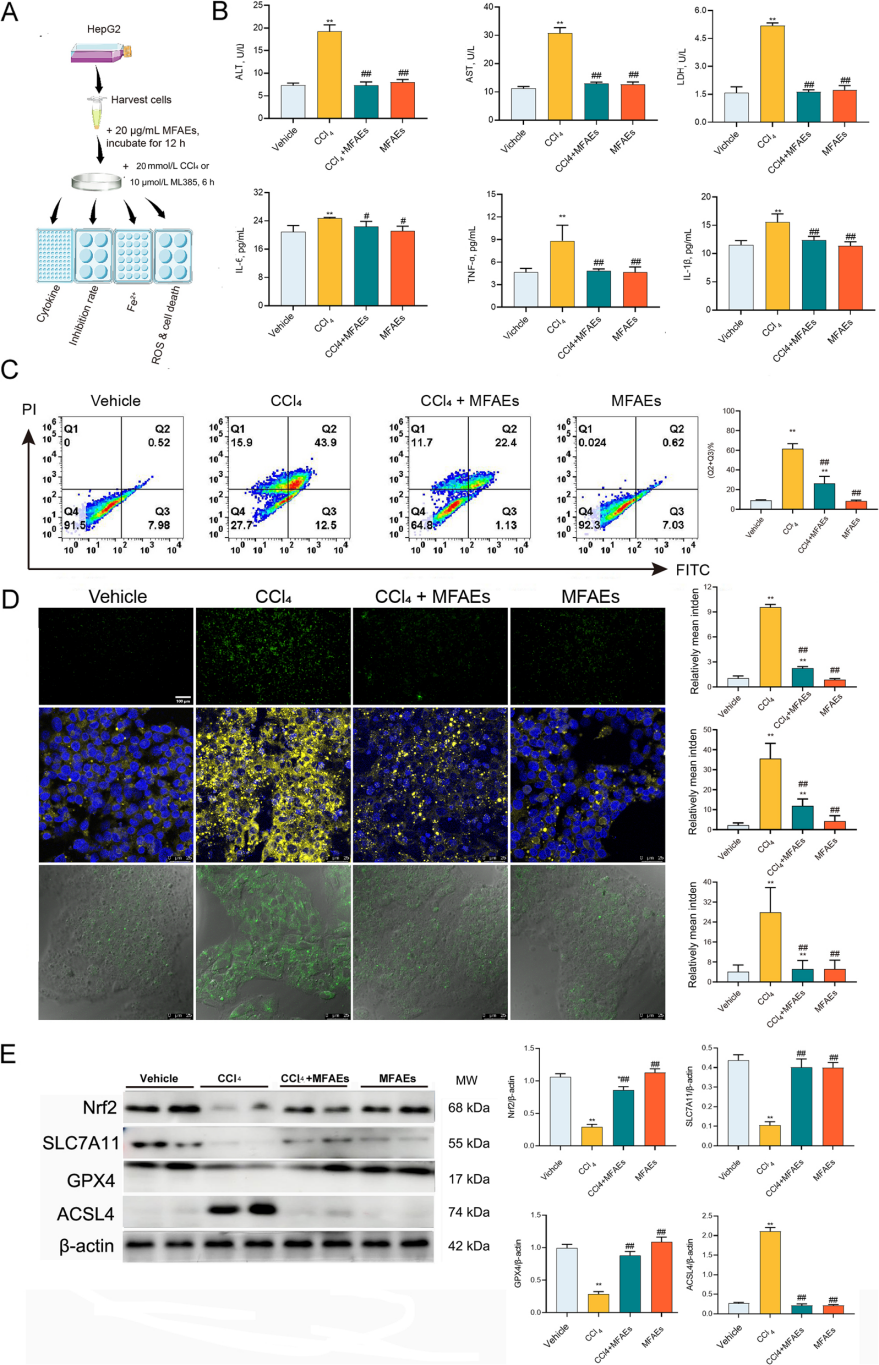
MFAEs downregulated Fe^2+^ accumulation and inflammation in vitro. **A** Outline of experimental procedures. **B** ALT, AST, LDH, IL-6, TNF-α and IL-1β levels in cell supernatants. **C** FITC/PI double staining results were detected by flow cytometry. *n *= 3 per group. **D** Representative image of immunofluorescence staining for ROS, Fe^2+^ and LPO in HepG2 cells. Bar, 100 μm (100×), 25 μm (640×). **E** Western blot analysis of Nrf2, SLC7A11, GPX4 and ACSL4. β-actin served as loading control. *Indicates a significant difference compared with the control group (*P < 0.05, **P < 0.01). ^#^Indicates a significant difference compared with the 10% CCl_4_ group (^#^*P* < 0.05, ^##^*P* < 0.01)

To further validate which step was regulated by MFAEs, we conducted an in vitro cell death assay (Fig. 7A). Hepatocytes are the primary targets of hepatotoxicity induced by CCl_4_. MFAEs significantly inhibited the activities of the liver functional markers AST, ALT and LDH and decreased proinflammatory cytokine IL-6, IL-1β and TNF-α levels induced by CCl_4_ (*P* < 0.01) (Fig. 7B). Moreover, 20 µg/mL MFAEs down-regulated apoptotic cells at early and late stages (*P* < 0.01) (Fig. 7C). In addition, ROS were also markedly decreased tin CCl_4_-treated cells and Fe^2+^ and LPO accumulation was also inhibited (*P* < 0.01) (Fig. 7D). Nrf2, SLC7A11 and GPX4 in vitro expression also increased while ACSL4 decreased in MFAEs-treated mice (*P* < 0.01)(Fig. 7E).

### MFAEs attenuated hepatocyte injury and ferroptosis via the Nrf2-dependent pathway

**Fig. 8:**
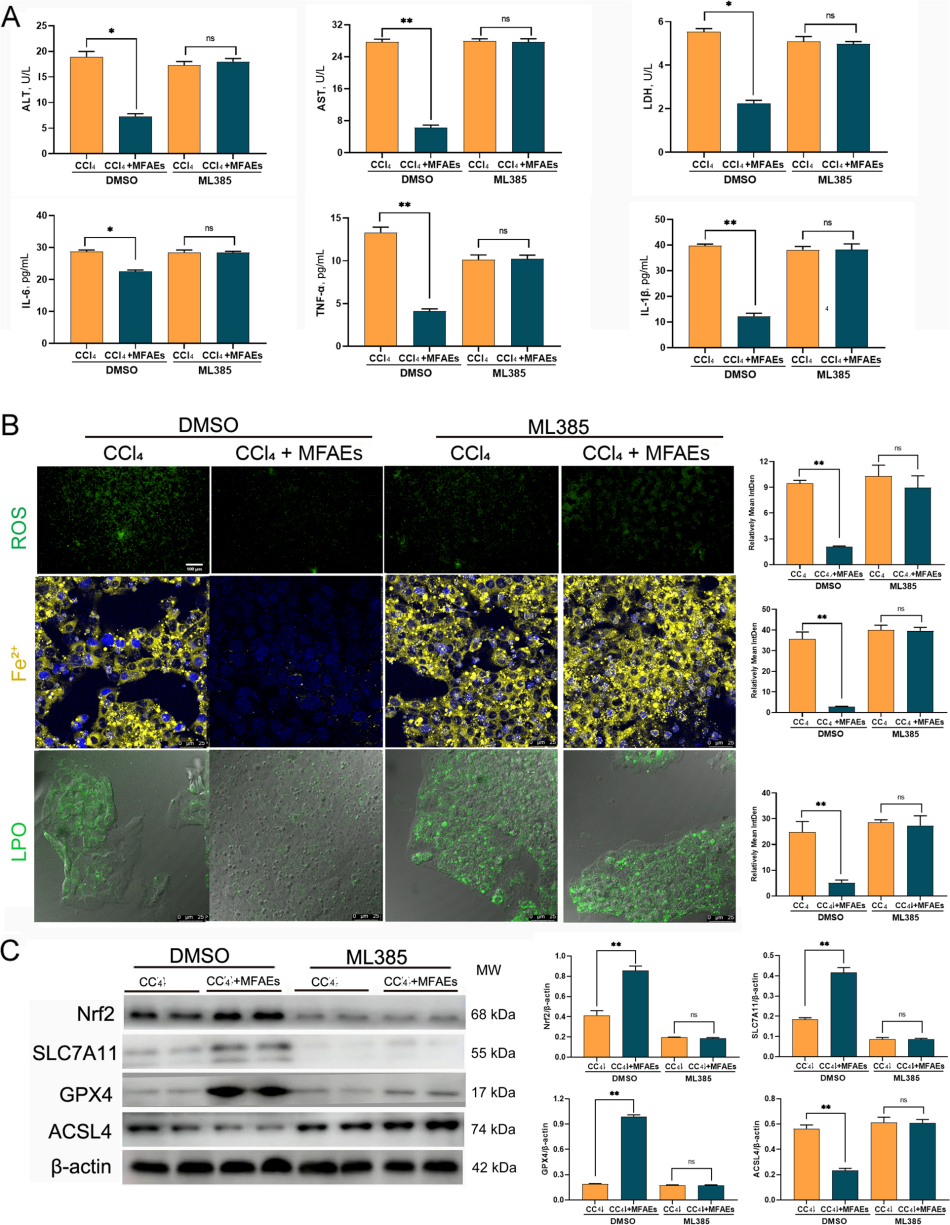
MFAEs attenuated hepatocyte injury and ferroptosis via the Nrf2-dependent pathway. **A** ALT, AST, LDH, IL-6, TNF-α and IL-1β levels in cell supernatants. **B** Representative image of immunofluorescence staining for ROS, Fe^2+^ and LPO in HepG2 cells. Bar, 100 μm (100×), 25 μm (640×). *n *= 3 per group. **C** Western blot analysis of Nrf2, SLC7A11, GPX4 and ACSL4. β-actin served as loading control. Results are expressed as mean ± SD. Independent sample t test was used for statistical analysis. ^*^ or ^**^indicates a significant difference between the two groups, ns indicates a nonsignificant difference between the two groups. ^*^P < 0.05, ^**^P < 0.01

Finally, to confirm whether MFAEs reduce ferroptosis and inflammation through the Nrf2 pathway, we used a selective Nrf2 inhibitor to further evaluate the protective effect of MFAEs on hepatocyte injury and iron death. MFAEs administration inhibited the increase of AST, ALT and LDH activities and the levels of pro-inflammatory factors IL-6, IL-1β and TNF-α induced by CCl_4_, these effects were reversed by supplementation with ML385 (*P* < 0.01) (Fig. 8A). The same reversal effect was observed for ROS production, Fe^2+^ accumulation in the cytoplasm and LPO distribution (Fig. 8B). In addition, the inhibitory effect of MFAEs on ferroptosis was eliminated after ML385 intervention (*P* < 0.01) (Fig. 8C).

## Discussion

The progression of acute or chronic liver injury is accompanied by liver fibrosis and the lack of timely relief will lead to a sharp increase in the probability of HCC [3]. The primary treatment methods to inhibit hepatofibrosis include liver vascular protection [31], inhibition of hematopoietic stem cell activation, extracellular matrix evolution and immune regulation [32, 33]. Due to the varying degrees of defects in various chemical synthetic drugs, there is no commercial drug to identify effective and low toxicity natural compounds that are commonly used for drug development and clinical medicine. In the present study, we revealed that MFAEs alleviated CCl_4_-induced Fe^2+^ accumulation and inflammation in HepG2 cells and effectively impeded 0.3% or 10% CCl_4_-induced acute/chronic liver injury progression in mice by regulating oxidative stress, inflammation and fibrosis. Mechanistically, MFAEs enhanced Keap1/Nrf2 signal transduction both in vitro and in vivo. Moreover, the hepatoprotective effects of MFAEs on Fe^2+^ accumulation and inflammation in hepatocytes and livers induced by 0.3% or 10% CCl_4_ were extensively abrogated under co-treatment with a Nrf2 inhibitor, ML385. Our data emphasized that MFAEs alleviated acute/chronic liver injury by activating Nrf2 targets to regulate lipid peroxidation, inflammation and fibrosis and that MFAEs can be applied as an underlining therapeutic strategy for acute/chronic liver injury.

CCl_4_-induced acute/chronic hepatic damage is characterized by hepatic lipid peroxidation, dysfunction, inflammation, fibrosis and liver injury and these effects are tightly linked to cirrhosis and oxidative damage [34]. We found that MFAE treatment suppressed lipid accumulation in CCl_4_-induced HepG2 cells and mitigated 0.3% and 10% CCl_4_ related hepatic damage, inflammation and fibrosis in mice suggesting that MFAEs resolved the major pathological consequences of liver fibrosis. These results were consistent with a previous study that used mulberrin as a regulator of ferroptosis that was responsive to Nrf2 signaling in hepatic fibrosis [35]. Furthermore, the systems pharmacology analysis clarified the potent action of MFAEs on liver fibrosis and the inflammatory response. Rather than focusing only on a disease model of liver fibrosis, a more comprehensive and systematic study of the role of MFAEs in acute/chronic liver injury was performed by establishing 0.3% or 10% CCl_4_ models in vivo and in vitro. Our data indicated that Keap1/Nrf2 signaling activation was required for MFAEs function in acute/chronic liver injury.

Nrf2 is the central player in the regulation of antioxidant molecules [36] and is critical in mitigating lipid peroxidation and ferroptosis [37]. Nrf2 is degraded by Keap1 and Keap1 inhibition allows Nrf2 to accumulate [38]. The Keap1/Nrf2 antioxidant-signaling pathway is a negative regulator of ferroptosis and SLC7A11 expression is an indicator of its activation [39]. The Nrf2 inhibitor ML385 reverses the regulation of MFAEs to CCl_4_-induced ferroptosis. However, Nrf2 suppression in itself does not promote hepatic fibroplasia although drugs that down-regulate Nrf2 in the fibrotic liver are able to inhibit further fibrosis [40, 41]. We found similar results and together these indicate that Nrf2 activation by MFAEs can function as a therapeutic target. The capacity of MFAEs to activate Keap1/Nrf2 signaling has also been validated in other pathological conditions (for example, alcohol-induced liver damage [19, 42, 43], cardiovascular disease [44], and antioxidant disorders [17, 45, 46].

At present, Nrf2/ARE signaling pathway, as a new target of anti-oxidative stress to achieve the goal of anti-liver fibrosis, has attracted more and more attention [47]. Several studies have confirmed that drugs can play an anti-hepatic fibrosis role by regulating Nrf2/ARE pathway. For example, Baicalein can regulate Nrf2/HO-1 and Bcl-2/Bax pathways to treat CCl_4_-induced liver fibrosis [48]; Quercetin ethyl acetate inhibits liver fibrosis in rats via decreasing the expression of α-SMA and TIMP1 depended on Nrf2 protein [49]; Ursolic acid can regulate Nrf2/ARE pathway, which can significantly prevent CCl_4_ induced hepatotoxicity and fibrosis [50]. Therefore, further study of the Nrf2/ARE pathway will explain the new mechanism of liver fibrosis. There are several potential mechanisms underlying MFAEs-activated Keap1/Nrf2 pathways. First, MFAEs directly bind to Nrf2 to enhance Nrf2 transposition into the nucleus. Second, since MFAEs activate Nrf2 upstream activators to increase its kinase activity or enhance its interaction with MFAEs, this could also result in net flux of Nrf2 into the nucleus. Third, the mitochondrial function and ROS content might be critically meaningful for Nrf2 activation [51–53]. Hence, MFAEs might alter mitochondrial antioxidant levels and subsequently enhance Nrf2 activation. We found that once Nrf2 signaling was activated, ferroptosis in liver was down-regulated and this was consistent with other studies that ferroptosis contributes to liver fibrosis and inflammation [54–57]. Moreover, one study has indicated that SLC7A11 is a transcriptional target of Nrf2 [58, 59]. Our data also found that MFAEs elevated the expression of SLC7A11. SLC7A11 is a key component of the Xct system [60]. Suppression of SLC7A11 leads to GSH depletion and increased ferroptosis. Thus, MFAEs may activate Nrf2 signaling and then weaken hepatic ferroptosis through the antioxidant system that involves SLC7A11, GPX4 and other antioxidant enzymes.

## Conclusions

In summary, our findings indicated that MFAEs ameliorate acute/chronic liver injury by promoting the nuclear transport of Nrf2 and provide convincing evidence that MFAEs may have therapeutic benefits in other hepatic disorders.

## Supplementary Information

**Additional file 1: Table S1.** Ishak scoring system for liver fibrosis. **Table S2.** Ishak scoring system for liver inflammation. **Table S3.** The antibody for western blotting. **Table S4.** The antibody for immunohistochemistry. **Table S5.** Primers for qRT-PCR. **Table S6.** Chemical composition identification in MFAEs by LC-Orbitrap-ESI-MS. **Fig. S1.** Chemical composition identification in MFAEs by UHPLC-LTQ-Orbitrap-MS spectrometry. **Fig. S2.** Analysis of interaction targets (A) and pathway enrichment (B-C) between MFAEs and liver injury. **Fig. S3.** Expression and liver pro-inflammation factors levels in acute/chronic liver injury mice.

## Data Availability

All relevant data of the current study can be requested from the authors.

## Abbreviations

ACSL4/Acsl4: Acyl-CoA synthetase long chain family member 4
BD: Bifendate
CCl_4_: Carbon tetrachloride
GPX4/Gpx4: Glutathione peroxidase 4
GSH: Glutathione
GSH-Px: Glutathione peroxidase
GST: Glutathione S-transferase
H&E: Hematoxylin and eosin
HA: Hyaluronic acid
HMGB1: High mobility group box 1
HO-1: Heme oxygenase 1
HYP: Hydroxyproline
IL-1β/Il-1b: Interleukin 1 beta
IL-6: Interleukin 6
iNOS: Inducible nitric oxide synthase 2
Keap1: Kelch-like ECH-associated protein 1
Lc3B: Microtubule-associated protein 1 light chain 3 beta
LDH: Lactate dehydrogenase
LPO: Lactoperoxidase
MDA: Malondialdehyde
MFAEs: Mori Fructus aqueous extracts
MMP1: Matrix metallopeptidase 1
MMP2: Matrix metallopeptidase 1
MMP9: Matrix metallopeptidase 9
NCOA4: Nuclear receptor coactivator 4
NF-κB: Nuclear factor kappa B
NQO-1/Nqo1: NAD(P)H dehydrogenase, quinone 1
Nrf2/Nfe2l2: Nuclear factor erythroid derived 2 like 2
POD: Peroxidase
ROS: Reactive oxygen species
SLC7A11/Slc7a11: Solute carrier family 7 member 11
TBA: Total bile acid
TBIL: Total bilirubin
TCMSP: Traditional Chinese Medicine Systems Pharmacology
TIMP1: Metallopeptidase inhibitor 1
TNF-α: Tumor necrosis factor alpha
T-SOD: Total superoxide dismutase
TUNEL: Transferase dUTP nick end labeling
VEGFA: Vascular endothelial growth factor A
α-SMA: Alpha-smooth muscle actin
γ-GT: Gamma-glutamyltransferase
