# Supplementary material for: Mori fructus aqueous extracts attenuates liver injury by inhibiting ferroptosis via the Nrf2 pathway
Source: J Anim Sci Biotechnol. 2023 Apr 10;14:56. doi: 10.1186/s40104-023-00845-0 (PMC10084661; doi:10.1186/s40104-023-00845-0)
Supplement: Supplementary file 1 — Additional file 1: Table S1. Ishak scoring system for liver fibrosis. Table S2. Ishak scoring system for liver inflammation. Table S3. The antibody for western blotting. Table S4. The antibody for immunohistochemistry. Table S5. Primers for qRT-PCR. Table S6. Chemical composition identification in MFAEs by LC-Orbitrap-ESI-MS. Fig. S1. Chemical composition identification in MFAEs by UHPLC-LTQ-Orbitrap-MS spectrometry. Fig. S2. Analysis of interaction targets (A) and pathway enrichment (B-C) between MFAEs and liver injury. Fig. S3. Expression and liver pro-inflammation factors levels in acute/chronic liver injury mice. [file 40104_2023_845_MOESM1_ESM.doc]

**Table S1** Ishak scoring system for liver fibrosis

| **Lesion degree** | **Score** |
| --- | --- |
| No fiber | 0 |
| Some portal areas have fibrous hyperplasia, with or without short fibrous septum | 1 |
| Most portal areas have fibrous hyperplasia, with or without short fibrous septum | 2 |
| Most of the portal areas have fibrous hyperplasia, and occasionally the portal areas are bridged by fibers | 3 |
| Fibrous hyperplasia in portal duct area with obvious fibrous bridging (between portal duct and portal duct and between portal duct and central vein) | 4 |
| Obvious bridging (portal tube to portal tube and /or portal tube to central vein) and occasional nodules (incomplete sclerosis) | 5 |
| Possible or definite cirrhosis | 6 |

**Table S2** Ishak scoring system for liver inflammation

| **Type** | **Lesion degree** | **Score** |
| --- | --- | --- |
| Focal (macular) lytic necrosis, apoptosis and focal inflammation | None | 0 |
|  | There is 1 necrotic area or less per 10× visual field | 1 |
|  | There are 2-4 necrotic areas per 10× visual field | 2 |
|  | There are 5-10 necrotic areas per 10× visual field | 3 |
|  | There are 10 necrotic areas per 10× visual field | 4 |
| Portal inflammation | None | 0 |
|  | Mild, partial or all portal areas | 1 |
|  | Moderate, partial or all portal areas | 2 |
|  | Possible or definite cirrhosis | 3 |
|  | Moderate / severe, all portal areas | 4 |
|  | Serious, all gate tube areas | 5 |

**Table S3** The antibody for western blotting

| **Antibody** | **CAT** | **Brand** | **Dilution** |
| --- | --- | --- | --- |
| MMP1 | bs-0424R | Bioss | 1:2000 |
| MMP9 | ab6671 | Abcam | 1:2000 |
| TIMP1 | bs-0415R | Bioss | 1:2000 |
| VEGFA | ab170099 | Abcam | 1:2000 |
| α-SMA | 14395-1-AP | Proteintech | 1:2000 |
| NF-κBp65 | ab32536 | Abcam | 1:1000 |
| NQO1 | bs-2184R | Bioss | 1:1000 |
| Nrf2 | 16396-1-AP | Proteintech | 1:1000 |
| Keap-1 | 10503-2-AP | Proteintech | 1:1000 |
| HO-1 | 27282-1-AP | Proteintech | 1:1000 |
| Ferritin/FTH1 | T55648 | Abmart | 1:1000 |
| HMGB1 | T55060 | Abmart | 1:1000 |
| GPX4 | T56959 | Abmart | 1:1000 |
| iNOS | ab213987 | Abcam | 1:1000 |
| SLC7A11/XCT | 26864-1-AP | Proteintech | 1:1000 |
| ACSL4 | 66617-1-Ig | Proteintech | 1:1000 |
| Lc3B | ab63817 | Abcam | 1:1000 |
| Atg5 | ab228668 | Abcam | 1:1000 |
| NCOA4 | 51114-1-AP | Proteintech | 1:1000 |
| Lamin B1 | P60054 | Abmart | 1:5000 |
| β-actin | ab8227 | Abcam | 1:5000 |

**Table S4** The antibody for immunohistochemistry

| **Antibody** | **CAT** | **Brand** | **dilution** |
| --- | --- | --- | --- |
| MMP1 | bs-0424R | Bioss | 1:100 |
| TIMP1 | bs-0415R | Bioss | 1:100 |
| VEGFA | ab170099 | Abcam | 1:100 |
| α-SMA | 14395-1-AP | Proteintech | 1:100 |
| NQO1 | bs-2184R | Bioss | 1:100 |
| Nrf2 | 16396-1-AP | Bioss | 1:100 |
| Keap-1 | 10503-2-AP | Bioss | 1:100 |
| HO-1 | 27282-1-AP | Bioss | 1:100 |

**Table S5** Primers for qRT-PCR

| **Gene** | **Primer sequence (5′→3′)** | **GenBank accession no** | **PCR product Tm, ℃** | **PCR product GC%** | **Product size, bp** |
| --- | --- | --- | --- | --- | --- |
| *Nfe2l2* | F: TTGGCAGAGACATTCCCATTTG  R: AAACTTGCTCCATGTCCTGCTCTA | NM_010902.4 | 77.6 | 51.7 | 172 |
| *Hmox1* | F: TGCAGGTGATGCTGACAGAGG  R: GGGATGAGCTAGTGCTGATCTGG | NM_001359638.1 | 79.4 | 57.6 | 144 |
| *Nqo1* | F: CAGCCAATCAGCGTTCGGTA  R: CTTCATGGCGTAGTTGAATGATGTC | NM_008706.5 | 76.2 | 52.1 | 117 |
| *Keap1* | F: AGCAGATCGGCTGCACTGAA  R: AGCTGGCAGTGTGACAGGTTG | NM_001110307.1 | 78.1 | 57.8 | 109 |
| *Il1b1* | F: TGGCAACTGTTCCTG  R: GGAAGCAGCCCTTCATCTTT | [NM_008361.4](https://www.ncbi.nlm.nih.gov/nuccore/NM_008361.4) | 75.7 | 52.4 | 105 |
| *Tnf-α* | F: CACGTCGTAGCAAACCACCAAGTGGA  R: TGGGAGTAGACAAGGTACAACCC | NM_001278601.1 | 75.5 | 52.5 | 140 |
| *Acsl4* | F: GAAAGGCTATGACGCCCCTC  R: ATCATGCGGACATTCCCTCC | XM_011247843.4 | 68.7 | 55.2 | 125 |
| *Slc7a11* | F: TGCCCGGATCCAGATTTTCC  R: CCAAGGGCAACCCCATTAGA | XM_017319590.3 | 70.2 | 54 | 152 |
| *Gpx4* | F: CCATGCACGAATTCTCAGCC  R: GGTGACGATGCACACGAAAC | NM_001367995.1 | 72.5 | 54.3 | 83 |
| *Gapdh* | F: TGTGTCCGTCGTGGATCTGA  R: TTGCTGTTGAAGTCGCAGGAG | NM_001289726 | 78.6 | 55.3 | 150 |

**Table S6** Chemical composition identification in MFAEs by LC-Orbitrap-ESI-MS

| **No.** | **Rt, min** | **Formula** | **Measured**  **[M-H]^−^** | **MS/MS (m/z)** | **Tentative identification** | **Structural formula** |
| --- | --- | --- | --- | --- | --- | --- |
| 1 | 1.66 | C_6_H_8_O_7_ | 190.9285 | 111.01; 87.01; 85.03; 191.02; 57.03 | Citric acid | 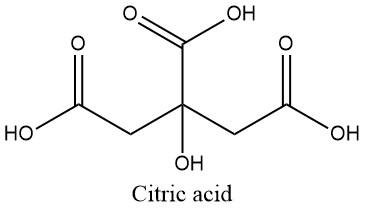 |
| 2 | 1.75 | C_6_H_8_O_7_ | 190.9285 | 111.01; 87.01; 85.03; 191.02; 57.03 | Citric acid | 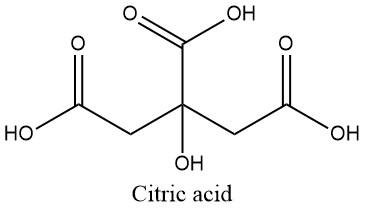 |
| 3 | 1.78 | C_9_H_8_O_4_ | 179.0351 | 59.01; 89.02; 179.06 | 4-Hydroxyphenylpyruvic acid | 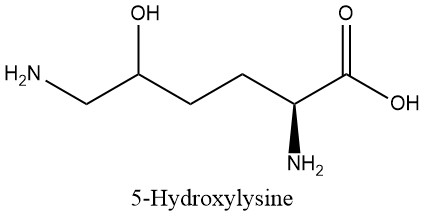 |
| 4 | 2.04 | C_6_H_14_N_2_O_3_ | 161.0455 | 73.03; 85.03; 131.03 | 5-Hydroxylysine | 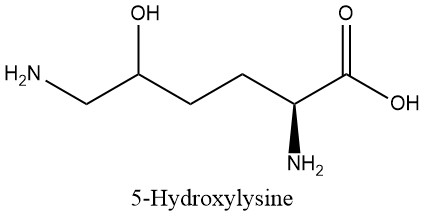 |
| 5 | 4.8 | C_7_H_6_O_4_ | 153.0193 | 109.03; 153.02; 108.02 | 2,5-Dihydroxybenzoic acid | 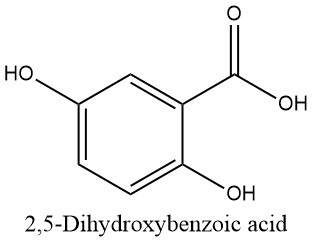 |
| 6 | 6.24 | C_16_H_18_O_9_ | 353.0727 | 191.06; 179.04; 135.05; 85.03; 111.05 | [Chlorogenic acid](javascript:treeMenu(0)) | 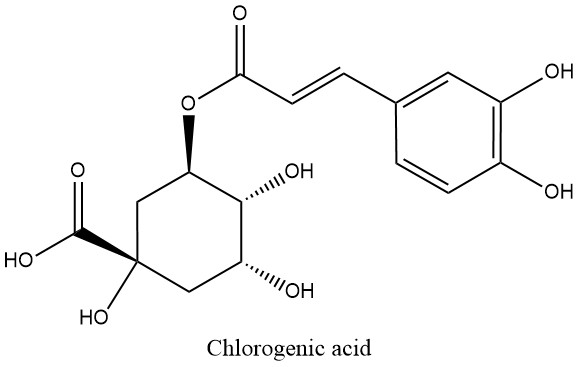 |
| 7 | 10.28 | C_16_H_18_O_9_ | 353.0727 | 191.06; 179.04; 135.04 | 3-O-Caffeoylquinic acid | 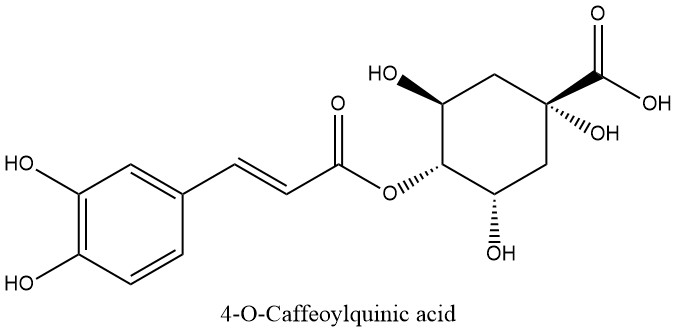 |
| 8 | 11.8 | C_16_H_18_O_9_ | 353.0883 | 173.05; 179.04; 191.06 | 4-O-Caffeoylquinic acid | 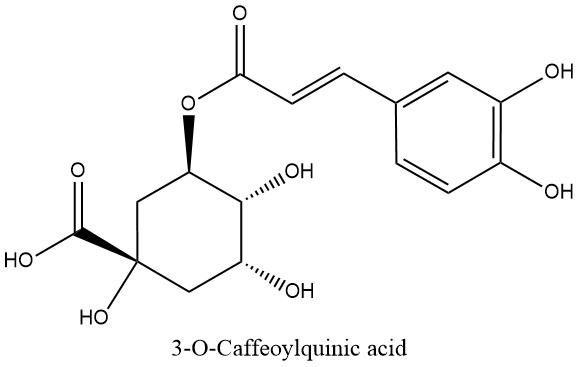 |
| 9 | 16.09 | C_21_H_21_O_12_ | 465.1028 | 285.04; 125.02; 303.5 | [DELPHINIDIN-3-GALACTOSIDE](https://pubchem.ncbi.nlm.nih.gov/compound/76311997) | 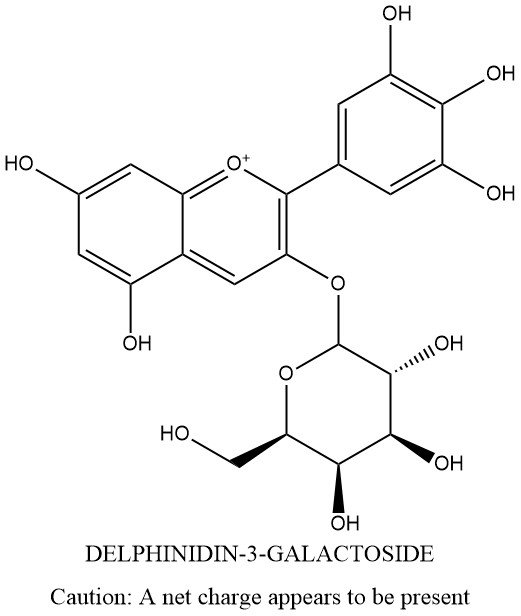 |
| 10 | 17.59 | C_7_H_6_Cl_2_O | 174.9561 | 118.9656; 132.9817; 174.9723 | 2,4-Dichloro-6-Methylphenol | 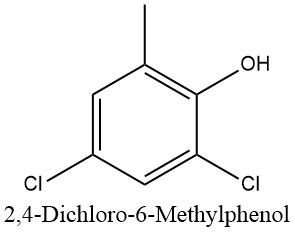 |
| 11 | 20.72 | C_27_H_30_O_16_ | 609.147 | 300.03; 301.04; 609.15 | Rutin | 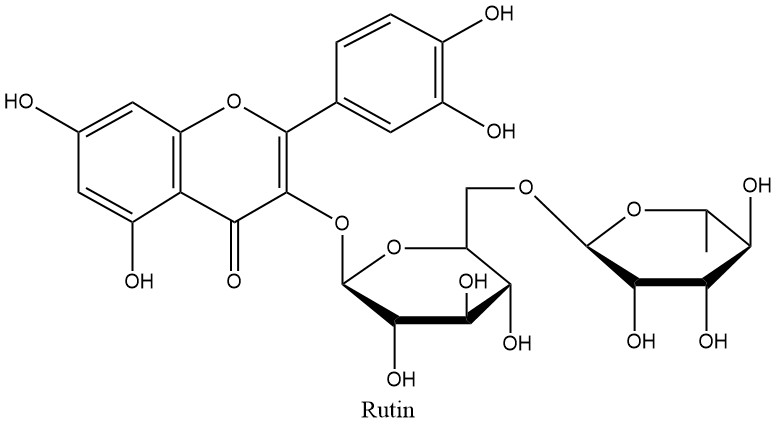 |
| 12 | 21.45 | C_21_H_20_O_12_ | 463.0885 | 300.03; 301.04; 463.09; 271.02 | [Hirsutrin](javascript:treeMenu(1)) | 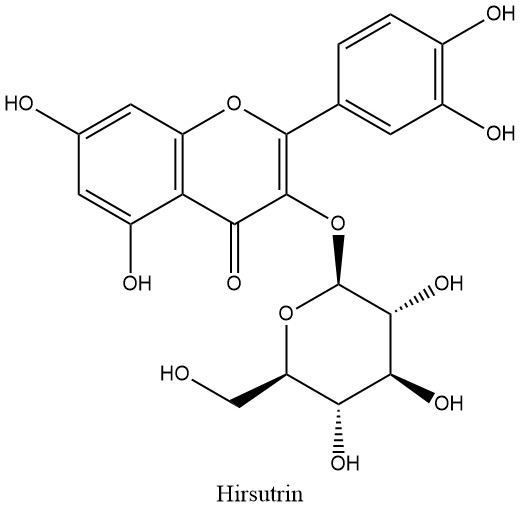 |
| 13 | 23.94 | C_18_H_22_O_5_ | 317.1972 | 59.01; 85.03; 155.11 | Zearalenone | 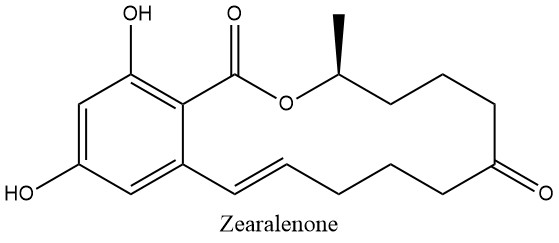 |
| 14 | 24.28 | C_21_H_21_O_11_ | 447.0939 | 285.04; 257.05 | Cyanidin-3-glucoside | 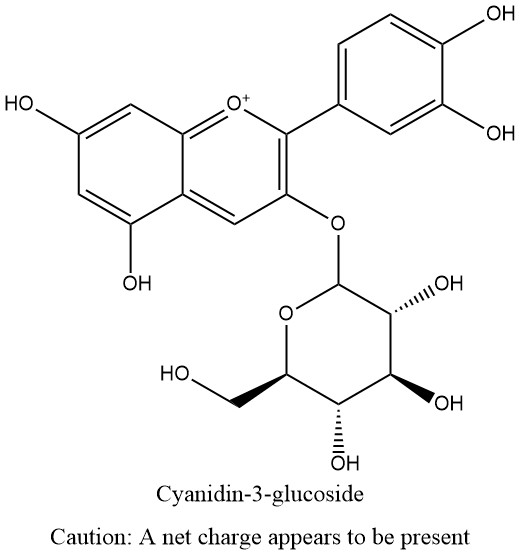 |
| 15 | 26.65 | C_15_H_10_O_7_ | 301.0356 | 301.04; 151.00; 179.00 | [Quercetin](javascript:treeMenu(0)) | 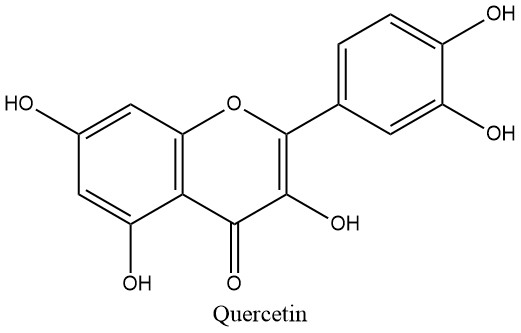 |
| 16 | 28.14 | C_10_H_8_O_3_ | 174.956 | 146.96; 174.96; 130.88; 118.97 | 4-Methylumbelliferone | 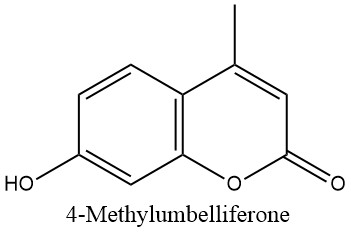 |
| 17 | 29.28 | C_21_H_30_O_3_ | 329.2336 | 329.23; 311.22; 273.82 | [17alpha-Hydroxyprogesterone](javascript:treeMenu(1)) |  |
| 18 | 31.15 | C_6_H_10_O_5_ | 160.8419 | 160.84 | 1,6-Anhydro-beta-D-glucose | 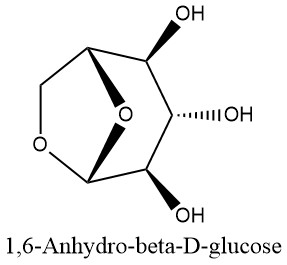 |


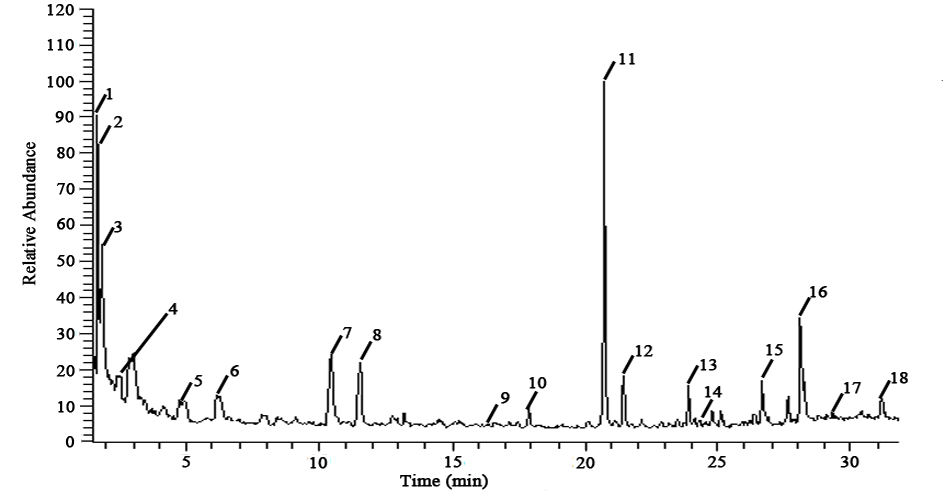


**Fig. S1** Chemical composition identification in MFAEs by UHPLC-LTQ-Orbitrap-MS spectrometry


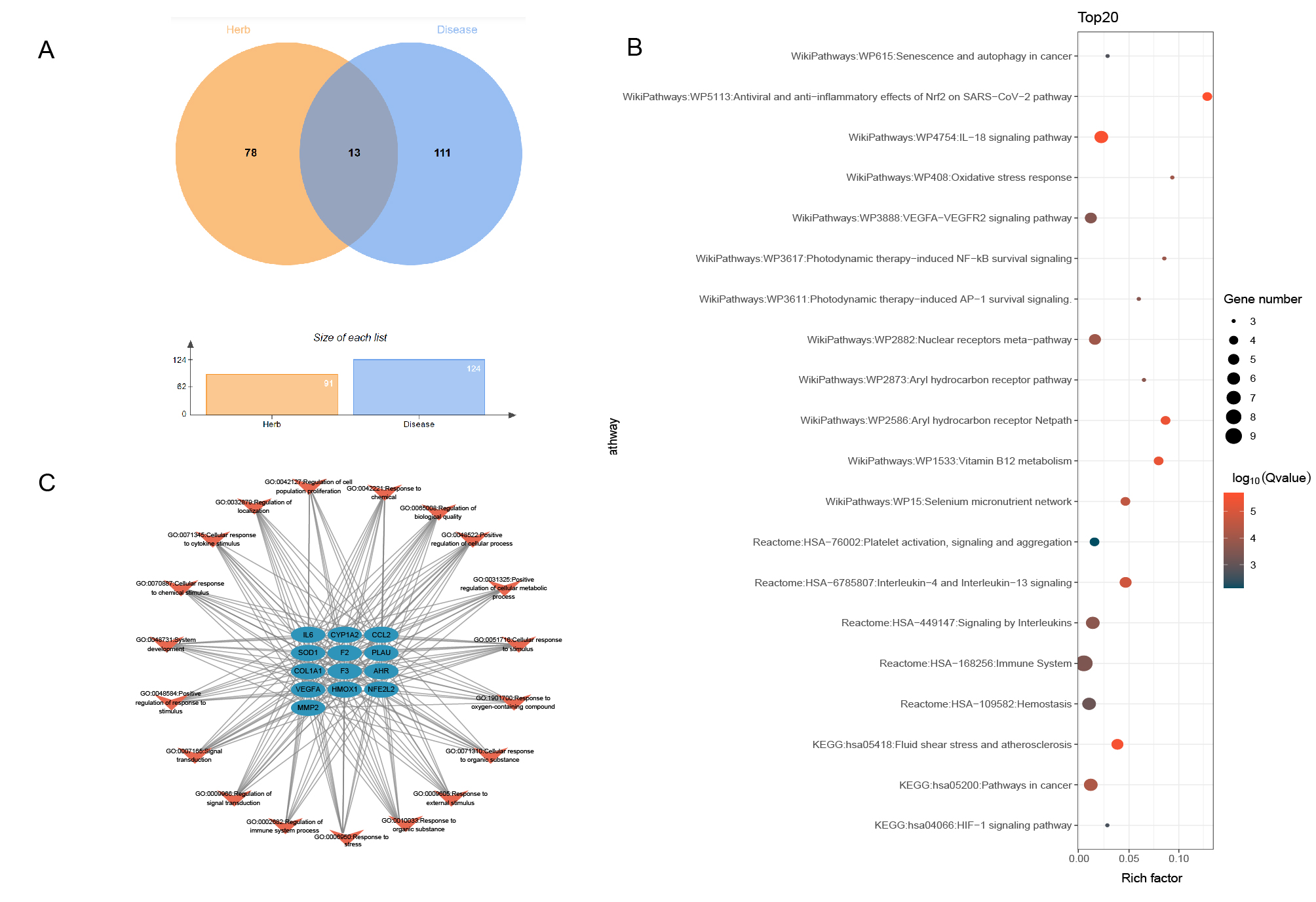


**Fig. S2** Analysis of interaction targets (A) and pathway enrichment (B-C) between MFAEs and liver injury


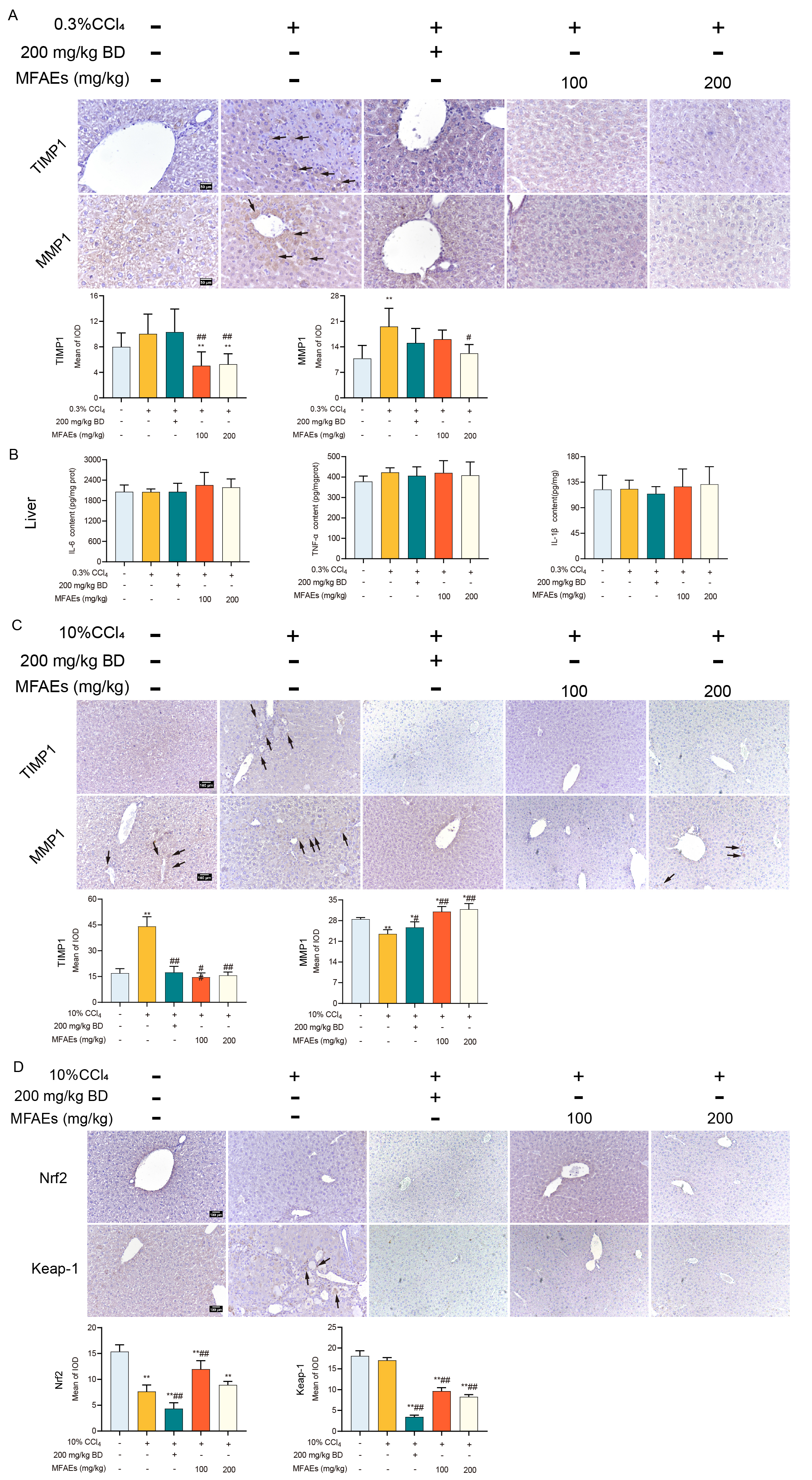


**Fig. S3** Expression and liver pro-inflammation factors levels in acute/chronic liver injury mice
